# Application of computer vision techniques for 3D matching and retrieval of archaeological objects

**DOI:** 10.12688/f1000research.127095.1

**Published:** 2023-02-16

**Authors:** Diego Jiménez-Badillo, Omar Mendoza-Montoya, Salvador Ruiz-Correa

**Affiliations:** 1 Museo del Templo Mayor, Instituto Nacional de Antropologia e Historia (INAH), Mexico City, CDMX, 06060, Mexico; 2 Instituto Tecnologico de Monterrey, Escuela de Ingenieria y Ciencias, Zapopan, Jalisco, 45201, Mexico; 3 Tecnologico de Monterrey, Escuela de Ingeniería y Ciencias, Monterrey, N.L., 64849, Mexico; 4 You-i Lab, Instituto Potosino de Investigacion en Ciencia y Tecnologia (IPICYT), San Luis Potosi, San Luis Potosi, 78216, Mexico

**Keywords:** 3D shape matching and retrieval, content-based shape engine, archaeological shape recognition

## Abstract

**Background**: As cultural institutions embark in projects oriented to digitise art and archaeological collections in three dimensions, the need for developing means to access the resulting 3D models has become imperative. Shape recognition techniques developed in the field of computer vision can help in this task.

**Methods**: This paper describes the implementation of three shape descriptors, specifically shape distributions, reflective symmetry and spherical harmonics as part of the development of a search engine that retrieves 3D models from an archaeological database without the need of using keywords as query criteria.

**Use case:** The usefulness of this system is obvious in the context of cultural heritage museums, where it is essential to provide automatic access to archaeological and art collections. The prototype described in this paper uses, as study case, 3D models of archaeological objects belonging to Museo del Templo Mayor, a Mexican institution that preserves one of the largest collections of Aztec cultural heritage.

**Conclusions**: This work is part of an ongoing project focused on creating generic methodologies and user-friendly computational tools for shape analysis for the benefit of scholars and students interested in describing, interpreting and disseminating new knowledge about the morphology of cultural objects.

## Introduction

Around the world, many professionals face the challenge of disseminating information of cultural heritage collections in such a way that objects can be known and studied, anywhere in the world, and preferably without the need of physical contact to guarantee their long-term preservation ( Ekengren *et al.*, 2021). To achieve that goal, cultural institutions have embarked on ambitious 3D digitisation projects and researchers have been looking for better means to improve access to the resulting 3D models ( Clark *et al.*, 2002; Ekengren *et al.,* 2021).

Digitisation indeed have been very successful thanks to the surprising evolution of photogrammetry and laser scanning, which makes possible to model the surface of objects with very little effort and in a relatively short period of time ( Pieraccini *et al.,* 2001). In fact, the continued adoption of these techniques has generated thousands, if not millions, of 3D digital models valuable for research and conservation. The geometric and morphological analysis of such models, for example, is now common in the cultural heritage field, as the bibliographic survey by Pintus *et al.* (2016) demonstrates.

Unfortunately, the search for better means to access collections has not achieved the same level of success. Many times, digital models are produced and then stored in a repository without implementing appropriate means to retrieve the 3D information ( Ekengren *et al.*, 2021; Koller *et al.*, 2009). Currently, the conventional way to locate models in a database consists of formulating a query by using keywords that describe the objects’ features; for example, the system is instructed to retrieve all 3D models corresponding to “tripod vessels” or “anthropomorphic figures”. However, this strategy works only if the categories used in the query are registered in the ontology and metadata built for that particular repository. For instance, if the term *bowl* does not exist in the database thesaurus, the search engine won’t find vessels that are similar but have been registered with another name. Another limiting factor is the language in which the objects are described, because the system might recognize “bowl”, but not *terrine* (French), *cuenco* or *cajete* (Spanish). Of course, these problems could be fixed using multilingual thesaurus and customized text-parsers, but such solution can hardly encompass all possible languages and semantic meanings of object’s descriptions. Additional problems may arise if the most relevant keywords to describe an object are unknown at the time of cataloguing the objects, or if important keywords to identify the objects are unknown to the users of the system.

In order to overcome these limitations and unlock the potential of 3D models for dissemination and research of cultural heritage objects, it would be better to have a system entirely focused on intrinsic visual characteristics of the objects, specifically a system that process queries analysing the shape of the objects without relying exclusively on keywords as search criteria. The development of such a system requires the computation of a numerical representations of shape for each object ( *i.e.* its shape descriptor), and the implementation of algorithms that compare all the shape descriptors stored in a database ( *i.e.* a matching operation) to facilitate the retrieval of 3D models ( Funkhouser *et al.,* 2003; Tangelder & Veltkamp, 2008).

This paper describes the first stage of an ongoing project oriented to such goal. It describes the implementation of a search-engine module, based on three types of shape descriptors and five dissimilarity measures that facilitate the matching and retrieval operations. The system, called ArcheoShape, will function as a kind of search engine that instead of using keywords will recognize objects automatically by comparing their computational ( *i.e.* numerical) shape descriptions. The benefits of this system are most clear in the context of museums, where it is necessary to find and retrieve 3D models from large collections.

## Basic requirements

**Figure 1.**
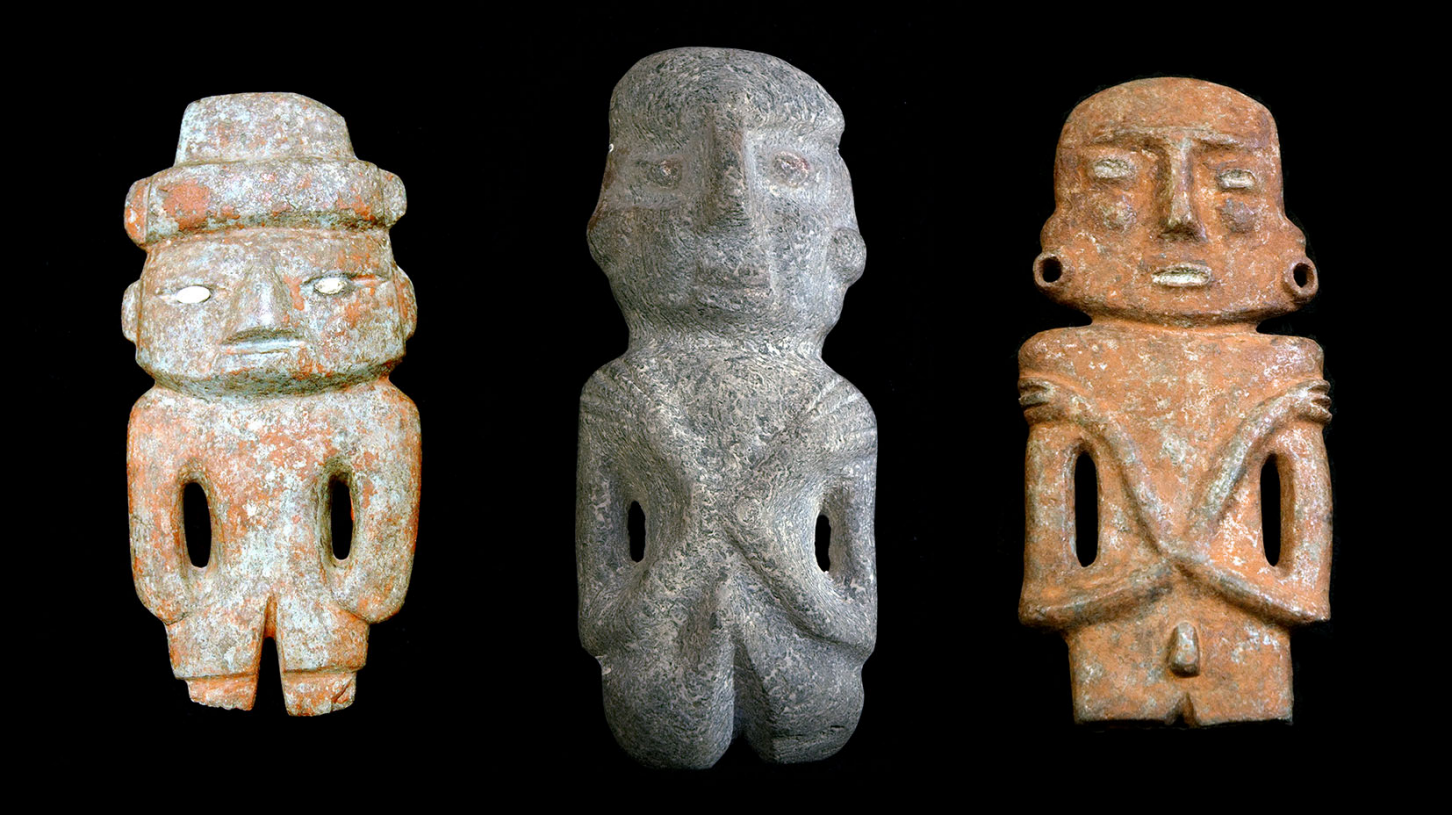
Three figurines found in the Sacred Precinct of Tenochtitlan. They are similar in their overall geometry, but differ in some details, such as the design of the head and the position of the arms. A recognition system must be able to find the 3D models of these objects, despite their partial differences in shape.

A system of shape recognition must be able to discover shape similarities between partially isometric objects, that is, between objects that share shape characteristics even if they are not identical. For example, a researcher might need 3D models of all the anthropomorphic figurines in a museum. In this case, the query should not be affected by the fact that one object lacks a head, while another is missing a leg. Also, it must retrieve several complete models of the same class, regardless of whether they differ in certain details ( Gal & Cohen-Or, 2006). The three objects shown in Figure 1 illustrate this situation; they belong to the same class of anthropomorphic figures, but their heads and arms show some morphological differences.

The second requirement is that the system be able to detect similarities without being affected by the variations of rotation, translation, reflection and scale of the 3D models. Ideally, different combinations of translation, rotation, and scale applied to equal objects during the digitisation process should not affect the system’s capacity to recognise their similarity.

To fulfil those requirements, it is necessary to apply a specialized set of methods of computer vision specifically designed to identify objects’ similarities efficiently, within the processing limits of today’s computers and that are sufficiently discriminatory to resolve the requirements of cultural heritage institutions.

## Development of a search-engine module

The development of shape recognition systems has been the subject of research since the 1980s, especially in the fields of computer vision, geometric modelling, and machine learning ( Besl & Jain, 1985; Bustos *et al.,* 2004, 2005; Campbell & Flynn 2001; Jain & Mishra 2014; Lara López *et al*., 2017; Loncaric 1998; Tangelder & Veltkamp 2008; Theologou *et al.*, 2014; Veltkamp & Hagedoorn, 1999). In the early 2000’s. Funkhouser *et al*. (2003) developed a system for retrieving 3D models applying the shape descriptor of spherical harmonics, implementing an interface that allowed queries by example and also sketch-based searches. Another early system was developed by Paquet and Rioux (2000) with shape descriptors based on tensors of inertia, distribution of normal vectors, distribution of cords and multiresolution analysis. Its interface allows entering a combination of parameters such as scale, shape or colour to search the database. Other innovations were due to Suzuki (2001) who included the processing of material data (colour and texture) as search criteria; although its descriptors require normalization of 3D models *via* PCA.

In the field of cultural heritage, Rowe *et al.* (2001) and Rowe and Razdan (2002) implemented a shape-based search engine for analysis and retrieval of native American ceramic vessels. Objects were modelled as parametric surfaces and the interface allows query by example and sketch-based query, and links to descriptive data. Another interesting system was designed by Schurmans *et al*. (2002) according to specific archaeological research objectives. For example, indicators of craft specialization can be gathered from the morphology of ceramic vessels. This involves matching shapes, as well as text, numeric, and vessel data calculated with the system tools.

Those projects demonstrate that the effectiveness of a recognition system depends above all on implementing efficiently two basic procedures. The first one consists in calculating a “shape descriptor”, that is a numerical representation of the form of each 3D model. Such descriptor can represent the global geometry of the object or a sample of its local features. The computation of shape descriptors involves a combination of mathematical, statistical, and more recently Machine Learning methods to represent shape in a numerical array or feature vector ( Tangelder & Veltkamp, 2008). Some examples of characteristics encoded by shape descriptors are the curvature or orientation of a certain quantity of patches drawn around points chosen in a random manner ( Jiménez-Badillo *et al.,* 2013), or alternately, signals calculated with spherical functions ( Kazhdan & Funkhouser, 2002; Kazhdan *et al.* 2003), reflective symmetry ( Kazhdan & Funkhouser 2002; Kazhdan *et al.*, 2004a), spin-images ( Johnson and Hebert, 1999), shape-contexts ( Mori *et al.,* 2001), histograms of spherical orientations ( Roman-Rangel *et al.*, 2016), and many others as described in bibliographic surveys by Bustos *et al*. (2004, 2005), Lara López *et al*. (2017), and Rostami *et al.* (2019).

The final objective is that the shape of the object is characterized in the best possible manner, as to constitute a “signature” (numerical representation) of the object readable by a computer. As mentioned above, the numerical descriptor must represent the shape regardless the object’s position, orientation and scale.

The second procedure consists in creating an index of the numerical representations of all the objects ( *i.e.* their shape descriptors), to facilitate the matching operation. The comparison between 3D models is done by measuring their degree of similarity with a mathematical function, such as Euclidean distance, that indicates the degree of their resemblance, so that when a query is implemented the system can rank objects from the more to the less similar ( Bustos *et al.,* 2004; Tangelder & Veltkamp, 2008).

The search-engine module developed over the course of this project is based on the implementation of three different global descriptors, namely shape distributions ( Osada *et al.,* 2002), reflective symmetry ( Kazhdan *et al.,* 2002, 2004a, 2004b) and spherical harmonic functions ( Kazhdan and Funkhouser, 2002; Kazhdan *et al.,* 2003).

As for determining the degree of dissimilarity between objects, five measures have been implemented: Euclidean distance, City block (Manhattan) distance, Chebychev distance ^1^, Minimum Coordinate distance, and Bhattacharyya distance. Each dissimilarity measure has been implemented in two norms: the probability density function (pdf), and the cumulative distribution function. Therefore, considering the quantity of descriptors and distance measures, the system offers in total thirty manners to calculate dissimilarity among pairs of 3D models ( Table 1).

**Table 1.** The five distance measures implemented in the prototype to calculate dissimilarity of shape descriptors.

Dissimilarity measure	Definition
**Euclidian distance**	$\sqrt{\sum_{i=1}^{n}{\left(x_{i}-y_{i}\right)}^{2}}$
**“City block” distance**	$\sum_{i=1}^{n}\left|x_{i}-y_{i}\right|$
**Chebychev distance**	$\max\left(\left|x_{i}-y_{i}\right|\right)$
**Minimum coordinate distance**	$\min\left(\left|x_{i}-y_{i}\right|\right)$
**Bhattacharyya distance**	$-\text{log}\sum_{i=1}^{n}\sqrt{\left(x_{i}y_{i}\right)}$

The following sections describe, in layman’s terms, the methods to compute the three shape descriptors selected for the implementation of the search-engine module.

### Shape distributions

The simplest shape descriptors included in the search-engine module are five probability distributions proposed by Osada *et al.* (2002). The names given to the descriptors depend on the type of calculation, “A” stands for angle and “D” for distance:

• A3: The angle between three random points on the surface of the 3D model.
• D1: The distance between the centroid of the model and one random point on its surface.
• D2: The distance between two random points on the surface.
• D3: The square root of the area of the triangle formed by three random points sampled on the surface.
• D4: The cube root of the volume of the tetrahedron formed by four random points sampled on the surface.

As Osada *et al.* (2002) recommend, we compute those variables for a very large sample of points selected from the surface-mesh of each 3D model, specifically 1,048,576 points ( *i.e.* 1024 × 1024 points). The measurements were then transformed into a frequency histogram (probability distribution), which could then be used as the global signature of the object’s shape. Once the shape histograms for all the objects had been computed, a normalization step was necessary to standardize the scales of all the histograms in order to avoid matching errors due to variations in the size of the objects. The objective is finding the scale that produces the minimal dissimilarity measure during the comparison of two object’s histograms. To achieve this, one of the methods proposed by Osada *et al*. (2002) involves the following steps: align both shape distributions ( *i.e.* histograms) so that the mean sample in each distribution equals 1; then find the minimum value *D*( *f* ( *x*), *sg* ( *sx*)) for values of log *s* from -10, 10, in 100 equally spaced intervals (where *f* and *g* represent the shape distributions of two models). Finally, select the minimum value among the results and use it as the dissimilarity measure for the two normalised shape distributions. This guarantees that two objects of the same shape but different sizes are recognized as similar, and vice versa, two objects of the same size but different shape are recognised as different.

The resemblance between any pair of objects can be determined by applying a function that measures dissimilarity between distributions ( *i.e.* histograms), for example Euclidean distance or any of the other measures mentioned in Table 1.

**Figure 2.**
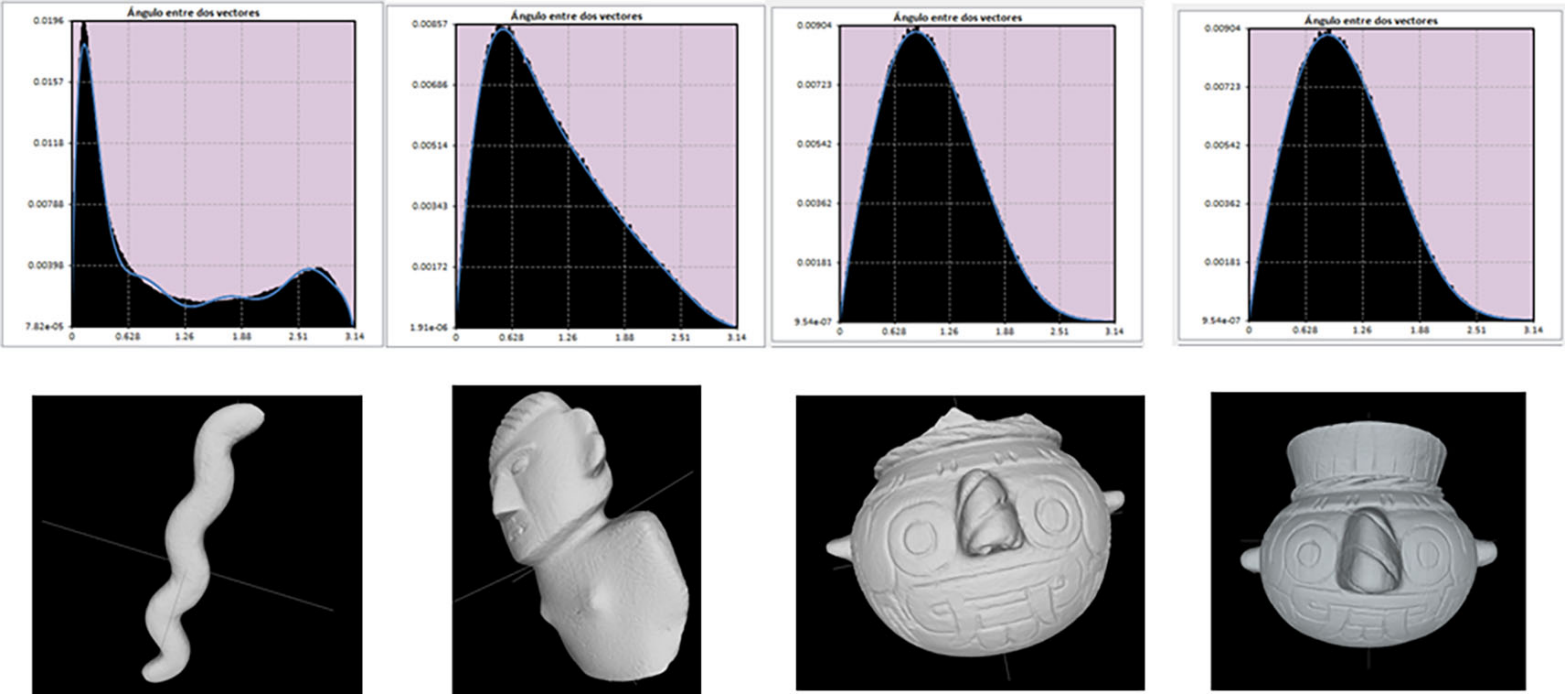
Frequency probability histograms resulting from obtaining measurements of angles between vectors for four objects from the Templo Mayor museum collection. Notice the great difference between the histogram of the serpentiform scepter (first object on the left), which shows the mode close to zero, and the histogram of the flat anthropomorphic figure (second from left to right). On the other hand, the great similarity of the histograms corresponding to the Tlaloc vessels located on the right side can be appreciated. Comparing these histograms allows the recognition system to determine whether or not two objects belong to the same class.

Figure 2 shows histograms resulting from the descriptor A3 (angle between three random points), representing the shape of four archaeological objects. Notice the probability distributions of the two models on the left, reflecting the differences between the long, wavy form of the serpentiform sceptre and the flat, wide anthropomorphic figurine. For an elongated figure, the angles between vectors tend to concentrate around the mode, while for flat objects the histogram would have a flat distribution because there would not be a predominant value of angle between vectors. In contrast, the images on the right correspond to two vessels whose histograms are quite similar because their shapes are also alike. Through this kind of comparison, the recognition system manages to identify similarities or differences between archaeological objects.

### Reflective symmetry descriptor

The second representation of shape, more complex but at the same time more effective, is the reflective symmetry descriptor proposed by Kazhdan *et al.* (2002, 2004a, 2004b). As these authors point out, symmetry —or the lack of it — is one of the most distinctive characteristics of any object.

Given a 3D model, denoted by function *g*, the concept of reflective symmetry implies that there is a reflection function γ , such that $g=\gamma \left(g\right)$ . This means that the pointwise distance between the points of surface *g* and the points of surface $\gamma \left(g\right)$ is zero *.*

Kazhdan *et al.* (2002) propose quantifying reflective symmetry with respect to several cutting planes, oriented on perpendicular axes that pass through the centroid of the model. For any given plane *P* cutting the shape *f*, the method consists in finding the function *g* such that g=γ with $⟦f-g⟧$ as small as possible *.* Mathematically this is expressed as:

$$\mathrm{SD}\left(f,\gamma \right)=\min_{g|\gamma \left(g\right)=g}⟦f-g⟧$$ (1)

where SD stands for Symmetry Descriptor. The more symmetric the shape *f* with respect to plane *P*, the smaller the value of $\left\|f-g\right\|$ *.* Large values of $\left\|f-g\right\|$ indicate that the surface is less symmetric.

**Figure 3.**
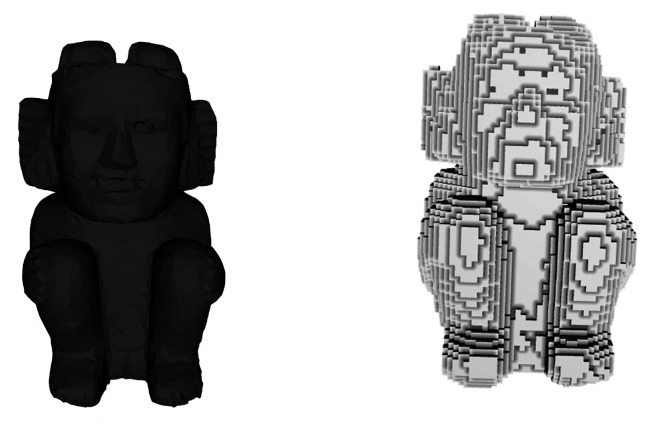
A 3D model of an archaeological figure and its voxel representation.

The calculation of reflective symmetry can be performed quicker and more efficiently by transforming the description of the surface mesh (3D model) into a discrete volumetric representation ( *i.e.* voxel grid). The process starts by immersing the triangular surface mesh inside a regular 3D grid. When a triangle of the mesh intersects a voxel of the 3D grid, such voxel is assigned a value of 1. Such rasterization process allows determining where the points and triangles of the mesh are located on the 3D grid. Notice that there are voxels in the grid that do not intersect the 3D mesh and therefore lack any information. For calculating the reflective symmetry descriptor, it is convenient to add to these voxels information related to how far they are from the surface of the model, for which the distance transform is used. This transform consists of assigning each voxel the distance to the nearest voxel belonging to the model. Then, the distance is transformed to a measure of similarity with the Gaussian function. Additional voxelization methods available can be found in Aleksandrov *et al*. (2021) and Huang *et al.* (1998). The resulting discrete representation consists of a 3D set of voxels, which appears like the archaeological model shown in Figure 3. ^2^

Once the voxel grid has been labeled in this way, the descriptor can be calculated. Broadly speaking, what it is done is to assume that when passing a cutting plane through the voxel grid there is perfect reflective symmetry between the two halves, so that one of the two halves can be replaced with the other if that property were fulfilled. Then, the reflective symmetry distance is calculated in the real model and compare it with the assumed model to measure any difference. If both representations are equal (zero distance), then there is perfect symmetry and a radius of 1 is assigned to the corresponding plane. If not, a value less than 1 is assigned according to how different these figures are.

**Figure 4.**
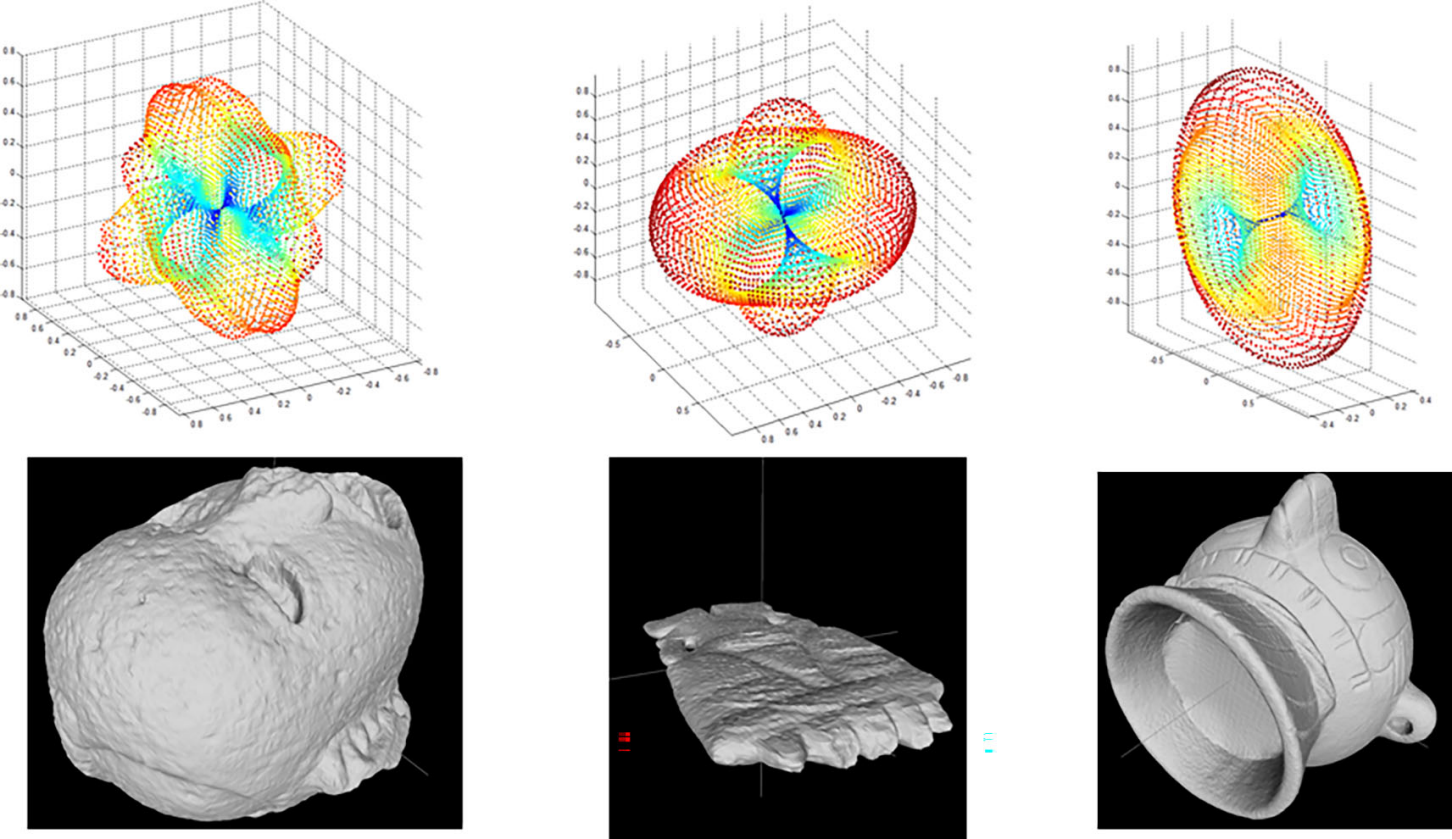
Graphs of the reflexive symmetry descriptors obtained for three objects from the Templo Mayor collection. As with the histograms shown in Figure 2, reflexive symmetry descriptors offer another method for calculating dissimilarity between 3D models.

Finally, the measures of symmetry obtained from a number of cutting planes ( *i.e.* axes of symmetry) are concatenated to generate a 3D graph, describing the model’s global symmetries. Values near 1 indicate perfect symmetry, while those near zero indicate that the two halves of a model are too asymmetrical. This graph is used to compare an object with any other for the matching and retrieval application. Visual representations of the descriptors obtained from three archaeological models are shown in Figure 4.

### Spherical harmonics descriptor

A third way of describing shapes on a computer is to consider them as outcomes of mathematical functions. Each stroke of a drawing, for example, can be regarded as a mix of 2D functions. The numerical representation of the whole drawing would be the sum of many functions. In practice, the functions are unknown, but they can be calculated by applying standard mathematical procedures such as the Fourier Transform, which would find the specific mix of simple functions that represent the complete drawing.

**Figure 5.**
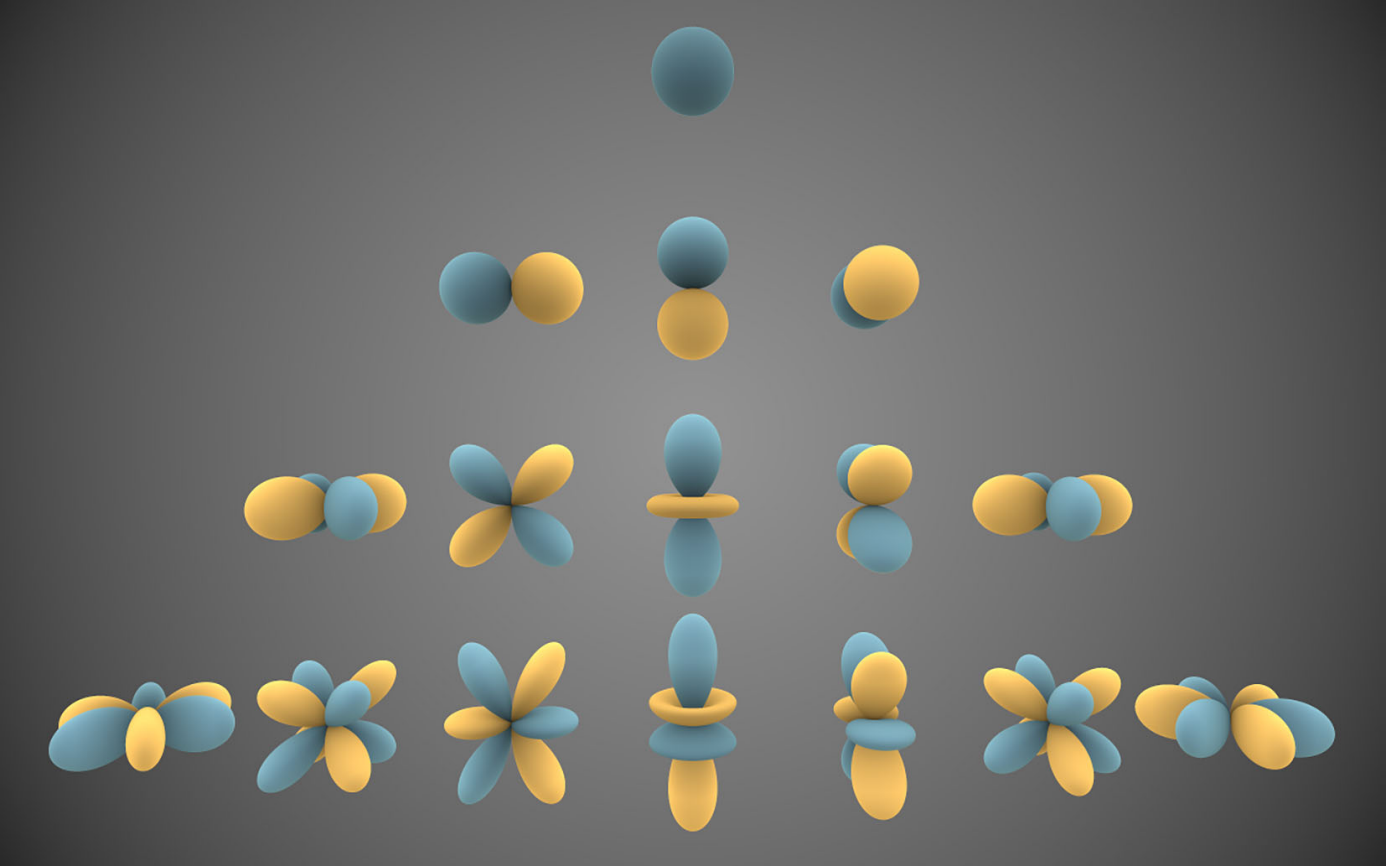
Graphical representation of harmonic functions calculated to obtain shape descriptors from 3D models. The simplest function is the sphere and from there higher order functions are derived. To obtain the mathematical description of a 3D model it is necessary to decompose the general form into constituent elements. The more complex the shape of an object, the more harmonic functions will be needed to describe it. The results of the decomposition are used to compare the shape of 3D models. Image by Inigo.quilez - Own work, CC BY-SA 3.0, (available here).

Something similar happens with 3D objects, but in that case the function describing the shape is defined on the surface of the sphere. One way of describing the shape of a surface is calculating the so-called spherical harmonic functions ( Figure 5). Intuitively, we can think of the spherical harmonic functions as “Lego” pieces that, together, help built the shape of complex 3D objects. This is possible, because mathematically speaking, spherical harmonics constitute a complete set of orthogonal functions and therefore form an orthonormal basis, upon which any function defined on the sphere (like the shape of a 3D model) can be expressed as the sum of these spherical harmonics.

The exact combination of spherical harmonic functions needed to describe a particular object can be found though a harmonic analysis. This method divides the complex surface of a 3D model into sums of relatively simple components.

Harmonic analysis is the branch of mathematics that deals with the problem of representing functions as the combination of basic elements called waves or harmonics. Typically, the term harmonic refers to functions with sinusoidal variations, but more strictly, it indicates any solution of Laplace’s Equation. The Fourier series is an example of a complete set of harmonics, which consists of sine and cosine waves of different frequencies.

In this work, we adopted the Spherical Harmonic Transform (SHT) to obtain a reduced representation of a 3D mesh. This method is a powerful tool for describing data on a sphere using spherical harmonics as basic functions. Given a function $f\left(\theta ,\varphi \right)$ in the spherical coordinates θ and φ , the decomposition of $f\left(\theta ,\varphi \right)$ in spherical harmonics $Y_{l}^{m}\left(\theta ,\varphi \right)$ is written as:

$$f\left(\theta ,\varphi \right)=\sum_{l=0}^{\infty }\sum_{\left|m\right|\leq l}c_{\mathrm{lm}}Y_{l}^{m}\left(\theta ,\varphi \right).$$ (2)

Here, l≥0 and m are integers such that $\left|m\right|\leq l$ , $c_{\mathrm{lm}}$ is the coefficient of the harmonic $Y_{l}^{m}\left(\theta ,\varphi \right)$ , and the general form of $Y_{l}^{m}\left(\theta ,\varphi \right)$ is:

$$Y_{l}^{m}\left(\theta ,\varphi \right)=\sqrt{\frac{2l+1}{4\pi }\frac{\left(l-m\right)!}{\left(l+m\right)!}}P_{l}^{m}\left(\cos\theta \right)e^{\mathrm{im\varphi}},$$ (3)

where $P_{l}^{m}\left(x\right)$ is a Legendre polynomial:

$$P_{l}^{m}\left(x\right)=\frac{{\left(-1\right)}^{m}}{2^{l}l!}{\left(1-x^{2}\right)}^{\frac{m}{2}}\frac{d^{l+m}}{{\mathrm{dx}}^{l+m}}{\left(x^{2}-1\right)}^{l}.$$ (4)

The problem in the Spherical Harmonic Transform is to calculate the coefficients $c_{\mathrm{lm}}$ .

In practice, it is not possible to calculate the coefficients of all the spherical harmonic functions. For this reason, we limit the order of the harmonics to a fixed value b (for instance 16 or 32) so that:

$$f\left(\theta ,\varphi \right)\approx \sum_{l=0}^{b}\sum_{\left|m\right|\leq l}c_{\mathrm{lm}}Y_{l}^{m}\left(\theta ,\varphi \right).$$ (5)

Finally, the coefficients $c_{\mathrm{lm}}$ are estimated by finding the least-squares solution to equation $\left(5\right)$ . That is to say, for a set of n points $\left\{\left(\theta_{1},\varphi_{1}\right),\left(\theta_{2},\varphi_{2}\right),\ldots ,\left(\theta_{n},\varphi_{n}\right)\right\}$ where $f\left(\theta ,\varphi \right)$ is evaluated, we calculate the values of the coefficients $c_{\mathrm{lm}}$ that minimize:

$$\sum_{i=1}^{n}{\left(f\left(\theta_{i},\varphi_{i}\right)-\sum_{l=0}^{b}\sum_{\left|m\right|\leq l}c_{\mathrm{lm}}Y_{l}^{m}\left(\theta_{i},\varphi_{i}\right)\right)}^{2}$$ (6)

To describe a 3D mesh using the Spherical Harmonic Transform, we define the function $f^{r}\left(\theta ,\varphi \right)$ as the intersection between the voxelized version of the 3D mesh and the sphere of radix r , both centered at the origin. The function $f^{r}\left(\theta ,\varphi \right)$ takes the value 1 only if the sphere intersects a voxel of the mesh at the point (θ,φ ), otherwise, this function is 0. The Spherical Harmonic Transform is applied to different radii so that the functions $f^{r}\left(\theta ,\varphi \right)$ are characterized by their corresponding harmonic coefficients.

The simplest harmonic function is the sphere, so if the object resembles a balloon, only one harmonic component of degree zero is enough for describing it. However, if the model has a more complex shape, then it is essential to calculate several higher order harmonic functions.

**Figure 6.**
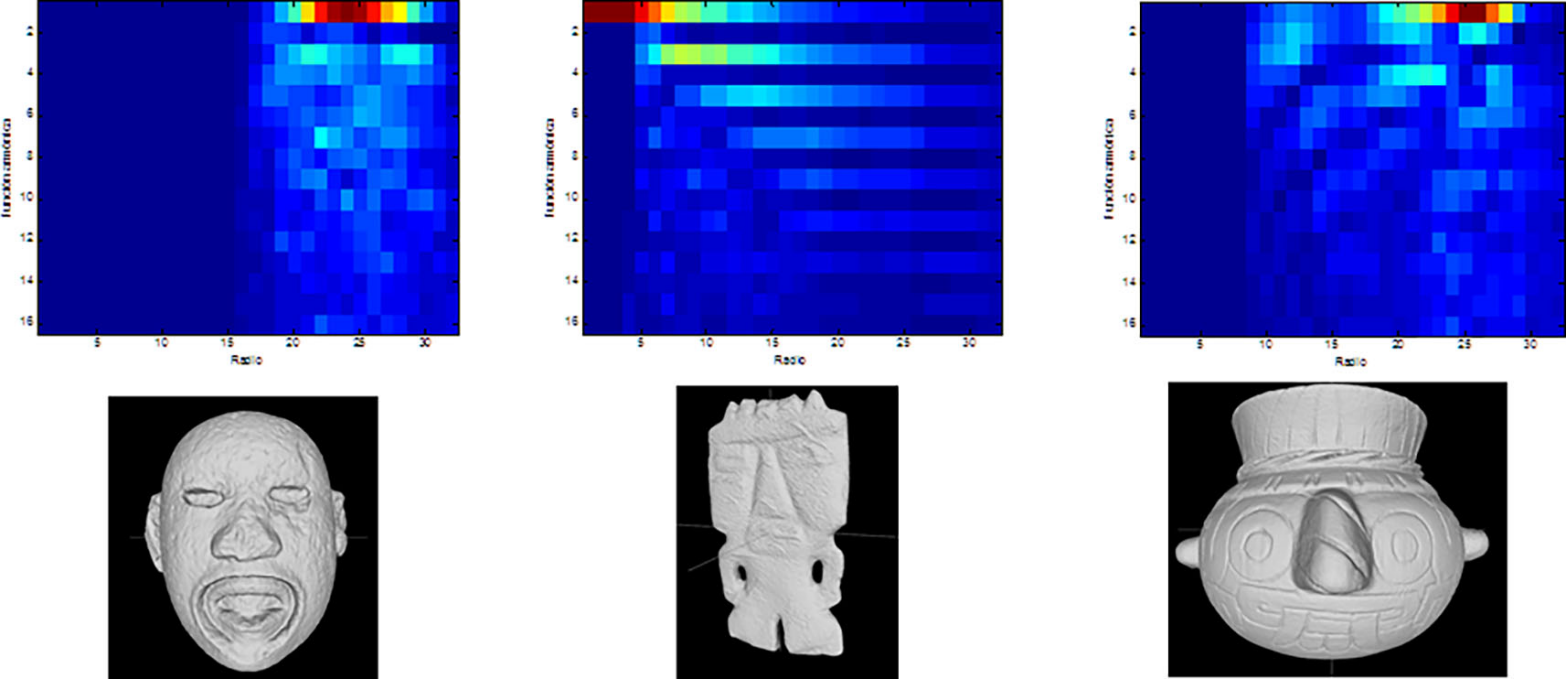
Graphs of the decomposition coefficients obtained by calculating harmonic functions for three objects of the collection of the Templo Mayor collection.

There are several methods to compute shape descriptors based on spherical harmonics. Some require a priori registration of the model along principal axes ( Saupe and Vranic 2001; Vranic & Saupe, 2001; Vranic *et al.,* 2001), but these are not good to process 3D models of the same class digitised with different orientations ( Funkhouser *et al.,* 2003). A method that solves that limitation is the one proposed by Kazhdan and Funkhouser (2002), and Kazhdan *et al.* (2003) and it is the one implemented during this project. In practice, the descriptor is computed as follows:

1. The 3D model is subjected to a voxelization process, like the one applied in the case of reflective symmetry (c.f. Huang *et al.* 1998). The size of the voxel grid is 64 × 64 × 64.
2. The 3D model is aligned with its voxel representation in such a way that is centre of mass coincides with the centre of the voxel grid.
3. A voxel is assigned a value of 1 if it contains any point on the surface of the 3D model, and 0 otherwise.
4. The voxel grid is decomposed into 32 spheres of radii 1 to 32, which produces 32 spherical functions.
5. Each sphere is decomposed as a sum of its first 16 spherical harmonics.
6. Finally, these different signatures are combined to obtain a 32 × 16 signature for the 3D model. The result is a 2D image that represents the decomposition coefficients for each harmonic function and each radii ( Figure 6).

To compare two objects using their harmonic representations, it is simply necessary to compute the Euclidean distance between them: “Thus, finding the K closest models to a query is equivalent to solving the nearest-neighbour problem” ( Kazhdan & Funkhouser, 2002). Figure 6 shows the spherical harmonics descriptor for three objects.

## User interface

Some early systems were tested with collections of 3D models produced with computer-aided design (parametric models) software and acquired on the internet. In contrast, we have developed a first module (mesh analyser) of a search engine called ArcheoShape that uses real archaeological objects. At this stage, the objective is to assess how good are the shape descriptors described in the previous sections to match and retrieve real archaeological objects, before deploying the entire system within a museum environment. The module developed over the course of this project is freely available in the following GitHub repository ( Mendoza-Montoya, 2023b), which contains the source code, written in C++, ready to be compiled in Windows and Linux, as well as an executable file. Instructions to compile are included in the GitHub repository. A sample of ten 3D models of archaeological artefacts are also provided under the Creative Commons Attribution-NonCommercial-NoDerivatives 4.0 International. These resources will allow any user to test the mesh-analyzer module in a local computer.

To test the implementation of the shape descritors, we used a sample of nearly 500 archaeological artefacts from the Museo del Templo Mayor. The collection is available for research purposes through specific agreements with Instituto Nacional de Antropología e Historia. ^3^

The Museo del Templo Mayor preserves objects discovered between 1978 and 1982 within the area occupied by the Sacred Precinct of Tenochtitlan, the most important religious centre of the Aztecs and nowadays a famous archaeological site adjacent to Zócalo square in Mexico City ( Matos Moctezuma, 1988). The core collection includes more than 8000 objects from ritual offerings found in the main pyramid temple ( *i.e.* Templo Mayor) of the site and its surroundings, and include ritual artefacts, flora, fauna, and human remains ( López Luján, 1994; Nagao, 1985). The collection has increased considerably in recent years thanks to the excavations conducted in the same site by different research teams led by archaeologists Barrera Rivera and Islas Domínguez (2018), Barrera Rodríguez, and López Luján (2012, 2019). Indeed, between 2012 and 2019, 43 new offerings, containing 13,925 artefacts and 35,648 samples of organic material have been reported. Digitization of this collection is still at a very early stage, but we have been able to acquire a sample of 495 objects, including stone-masks, anthropomorphic and zoomorphic figures, clay vessels (bowls, pots, jars, braziers), religious paraphernalia like sceptres, earplugs, ritual pendants, as well as flint sacrificial knifes, flutes, and models of drums made of clay or stone. The implementation of the prototype was divided into two independent offline and online jobs. The main offline task consists in computing the shape distributions, reflective symmetry and spherical harmonics descriptors of all the archaeological 3D models available.

**Figure 7.**
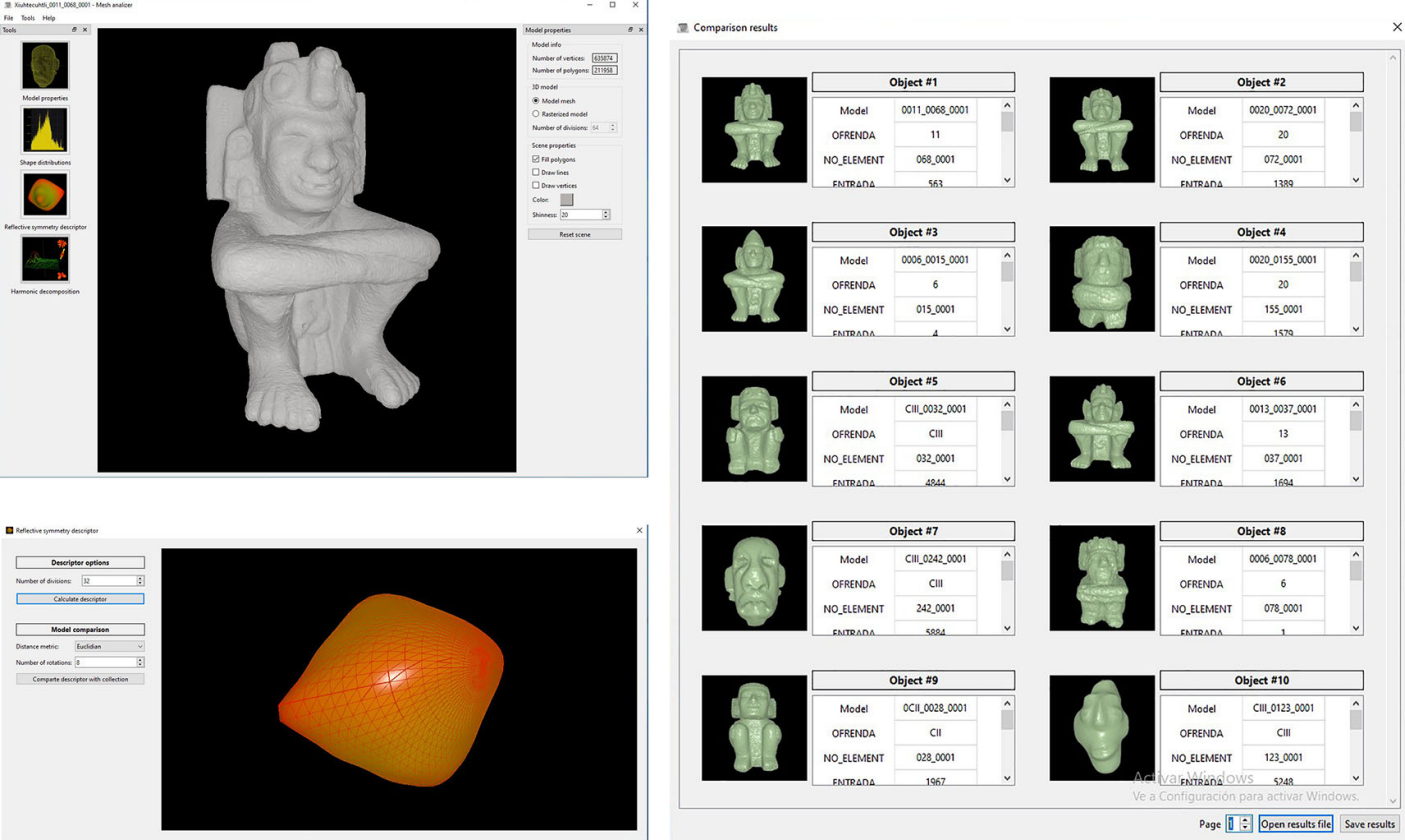
Illustration of the software developed for the search and recovery of 3D models of archaeological objects. Above, on the left, the consultation model ( *i.e.* anthropomorphic sculpture) is shown; below left illustrates obtaining the reflexive symmetry descriptor for that query model; on the right are the search results obtained when the user requests to compare the query model with the models stored in the repository. It can be seen that the system retrieves all objects similar to the query model.

The actual matching and retrieval operations are done online through the following steps:

• First step. Through an open-file window, the user enters a query model consisting of a point cloud or surface mesh ( *i.e.* query model) as an example of the class of objects he wants to retrieve from the database. For example, an anthropomorphic figure like the one shown in Figure 7. This step avoids the input of keywords as search criteria.
• Second step. The user selects the shape descriptor (shape histograms; reflective symmetry; or spherical harmonics) to apply it to the query model. There are no rules to select an algorithm, it is expected that the user tries different options to see which one works best for a specific set of archaeological objects.
• Third step. The system compares the shape descriptor of the query object with the shape descriptors of all 3D models stored in the repository. The search engine calculates the distance between the shape descriptor of the query model and the descriptors of all the other models stored in the database.
• Fourth step. The models that match the shape of the query model are ranked from the more to the least similar.
• Fifth step. Finally, the system presents the results on the screen sorted from the most to the least similar. There is no limit on the number of results displayed by the system.

**Figure 8.**
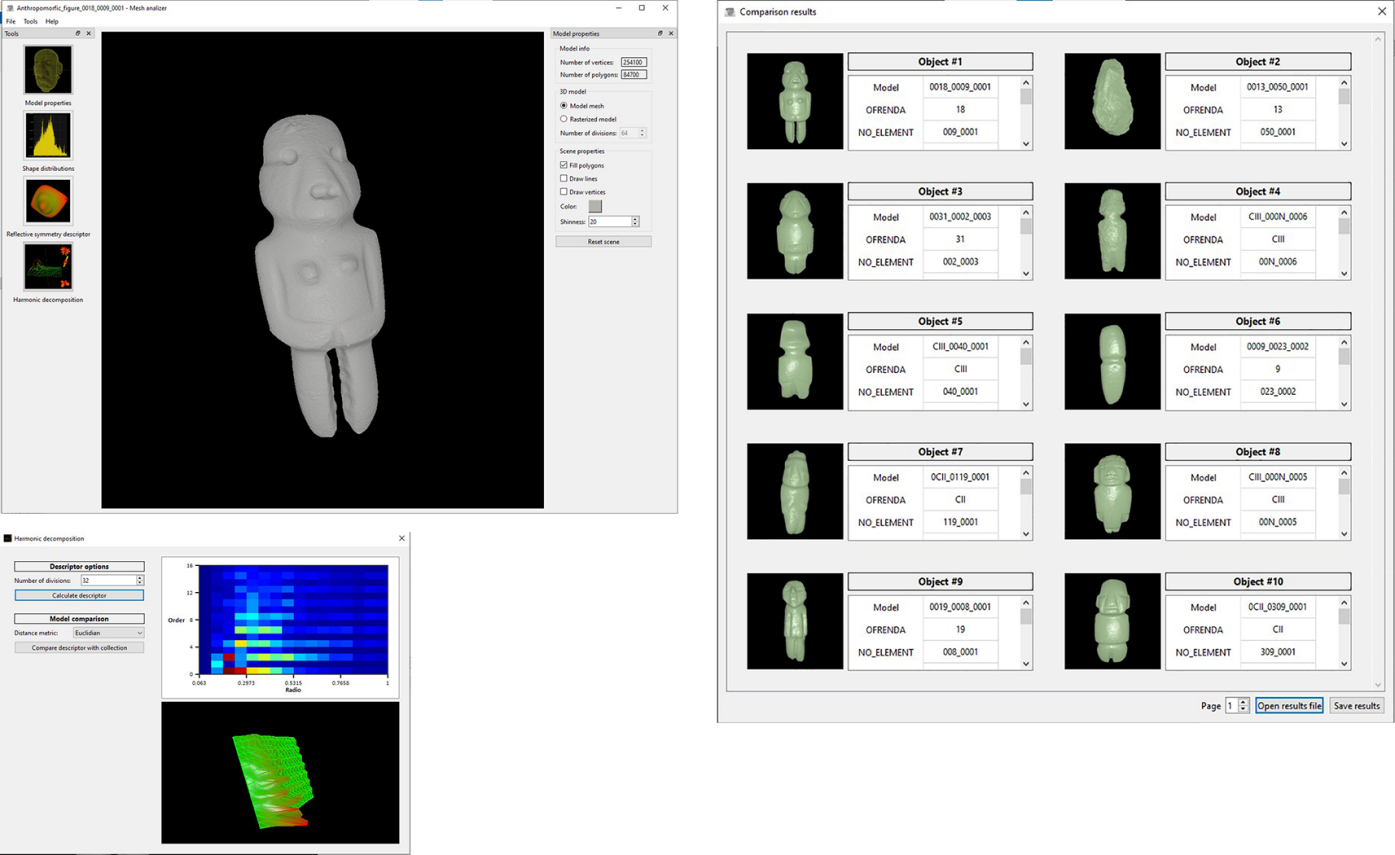
Another example of searching and retrieving 3D models. In this case, all copies of anthropomorphic figures were requested from the system applying the descriptor of harmonic functions.

**Figure 9.**
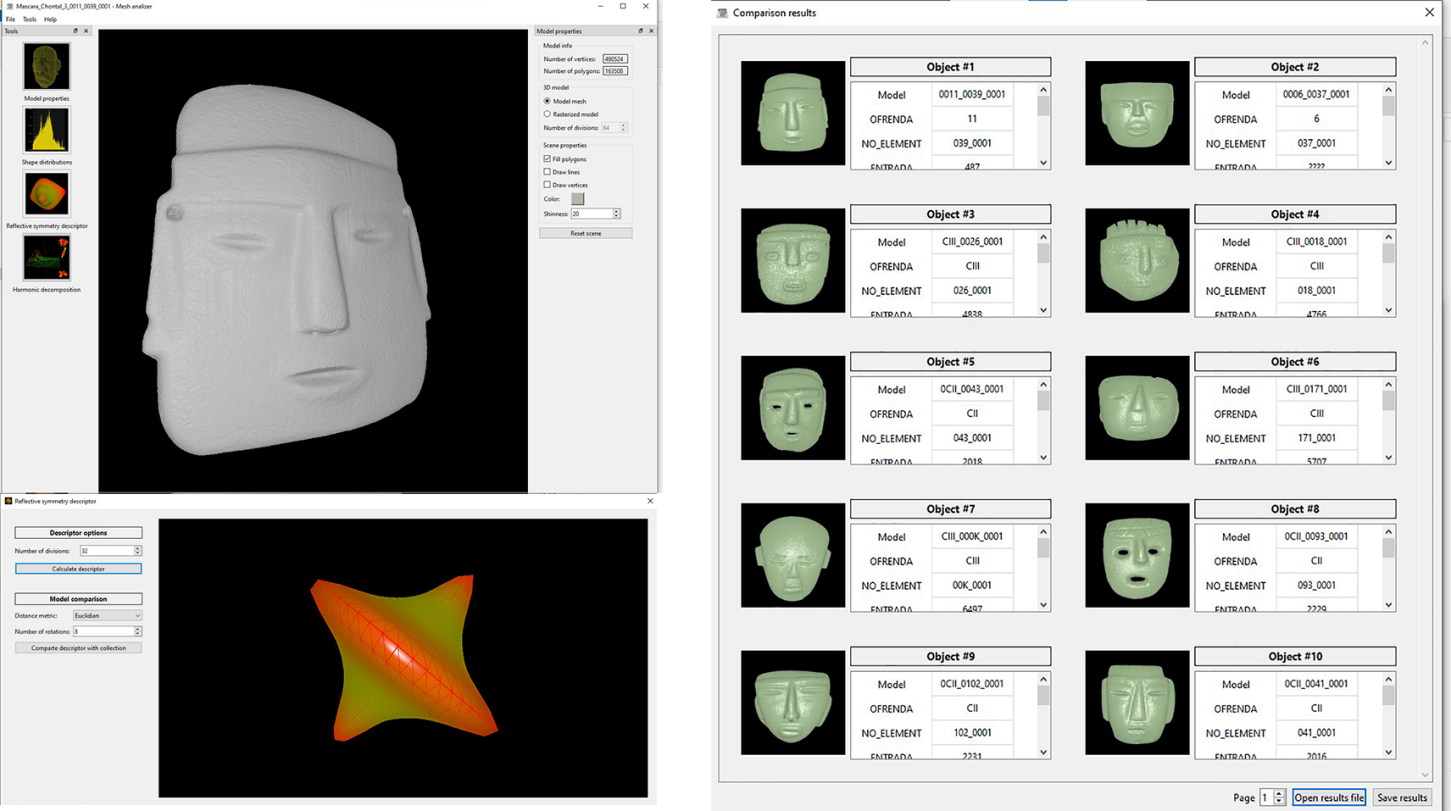
A third example of how the software works. In this case stone masks were recovered. It should be noted that, despite the fact that the query model lacks a fragment, the system was able to produce the expected results, even recovering a mask fragment that clearly belongs to the class of the objects that the user expected.

Figures 7, 8 and 9 illustrate the user interface during three query examples. The first window (top-left) shows the query model. The icons on the left side allow the user to select one of the three shape descriptors that have been implemented, while several options on the right allow the user to change the appearance of the model ( *e.g.* visualise the query models as point cloud, triangular mesh or solid volume, or alter the colour). In the next window (bottom-left), the user can set the parameters necessary for calculating the shape descriptor of the query model, particularly the distance measure applicable for the matching operation. It is worth noticing that the system offers predefined parameters that work in all cases, but the user can set them to follow his/her own preferences. In choosing the predefined parameters, the main criteria were to ensure that the system had good shape recognition power in a short computation time. Once the user presses enter, the search engine proceeds to compare the query model with the descriptors of the models stored in the database and retrieve the results, which are shown in the window illustrated on the right-hand side of the figures.

## Conclusions

Computer tools for shape matching and retrieval designed specifically for archaeological research could improve access to collections in museum institutions. The development of ArqueoShape is a step forward in this direction.

The search-engine module developed over this project is generic, so we expect they would prove helpful in other contexts. The capacity of our system to perform matching and retrieval of real archaeological objects through the application of shape distributions, reflective symmetry, and spherical harmonics descriptors is significant. However, an extra module to provide full database capabilities to store, update and edit the 3D models are still under construction. Also, we expect to perform a benchmark analysis, whose results will be published shortly. Particularly important for further development is the implementation of additional shape descriptors that target local features, since these would help to refine the queries to specific details on the objects geometry.

We plan to embed the search-engine module described here into a web platform which will be organized around three main application channels:

1. The first channel would be a service platform for the automatic recognition, analysis, and classification of cultural heritage objects based on morphology. Any user can upload a collection of 3D models to have it analysed with the software tools developed throughout the project. For this operation, the user will not need any knowledge of Computer Vision or Machine Learning because all necessary software will be accessible through a very easy-to-use interface.
2. The second channel called research will be designed to encourage specialized collaboration between experts in Computer Vision, Machine Learning, and shape analysis interested in developing new algorithms, applications, and tools for morphological analysis of cultural heritage. Including our current deep learning applications for shape analysis and retrieval. This collaboration will facilitate access to papers, project proposals, discussion forums, and source code. New solutions to technical problems will be expected to evolve from this site. For example, one pervasive challenge when applying machine learning to archaeology is the lack of enough data to train automatic learning models. This channel could provide a forum for discussing new solutions, such as conditions for applying transfer-learning techniques to train models with external knowledge.
3. The third channel will be named People Interaction. Through this channel, scholars, students, and anyone interested in the project can establish collaboration for future projects and share data and resources from all over the world. The main objective is to create synergy to facilitate access to new 3D digital collections and to define new initiatives of morphological analysis with applications to archaeology and the Humanities.

## Data Availability

Zenodo: omendoza83/ArcheoShape-Data: ArcheoShape 0.2. https://doi.org/10.5281/zenodo.7591490 ( Mendoza-Montoya, 2023a). This project contains the following underlying data:
- Models. (10 triangular meshes of Aztec objects). - Resources. (6983 numerical shape descriptors, computed from 495 archaeological objects). - Icons. (Images for the user interface). - Screenshots. (Images of 495 archaeological objects, used to present results at the end of a search and matching operation). Data are available under the terms of the Creative Commons Attribution-NonCommercial-NoDerivatives 4.0 International (CC-BY-NC-ND 4.0).

## Footnotes

^1^
Also known as the L∞ metric, Chevychev distance determines which distance between two vectors is the greatest of their differences along any coordinate dimension.

^2^
The voxel grid preserves the topological characteristics of the original 3D model. These include connectivity, separation, coverage, and tunnelling. Connectivity registers the way voxels must be linked to each in order to preserve the specific shape of the object. Separation registers empty spaces and how these interact in the voxelised object. Coverage, register how “thin” or thick” the voxelised object is. Tunnelling registers the penetration of two voxelised elements of the object, that is how two or more parts of the object overlap and/or penetrate into each other ( Aleksandrov *et al*., 2021).

^3^
The authors can provide details of the legal and administrative procedures involved in sharing the collection.

## Software availability

Source code available from: https://github.com/omendoza83/ArcheoShape/tree/v0.3.0-alpha

Archived source code at the time of publication: https://doi.org/10.5281/zenodo.7583722 ( Mendoza-Montoya, 2023b)

License: MIT
