# Application of computer vision techniques for 3D matching and retrieval of archaeological objects

**DOI:** 10.12688/f1000research.127095.2

**Published:** 2024-03-25

**Authors:** Diego Jiménez-Badillo, Omar Mendoza-Montoya, Salvador Ruiz-Correa

**Affiliations:** 1 Museo del Templo Mayor, Instituto Nacional de Antropologia e Historia (INAH), Mexico City, CDMX, 06060, Mexico; 2 Tecnologico de Monterrey, Escuela de Ingeniería y Ciencias, Monterrey, N.L., 64849, Mexico; 3 Instituto Tecnologico de Monterrey, Escuela de Ingenieria y Ciencias, Zapopan, Jalisco, 45201, Mexico; 4 You-i Lab, Instituto Potosino de Investigacion en Ciencia y Tecnologia (IPICYT), San Luis Potosi, San Luis Potosi, 78216, Mexico

**Keywords:** 3D shape matching and retrieval, content-based shape engine, archaeological shape recognition

## Abstract

**Background:**

As cultural institutions embark in projects oriented to digitise art and archaeological collections in three dimensions, the need for developing means to access the resulting 3D models has become imperative. Shape recognition techniques developed in the field of computer vision can help in this task.

**Methods:**

This paper describes the implementation of three shape descriptors, specifically shape distributions, reflective symmetry and spherical harmonics as part of the development of a search engine that retrieves 3D models from an archaeological database without the need of using keywords as query criteria.

**Use case:**

The usefulness of this system is obvious in the context of cultural heritage museums, where it is essential to provide automatic access to archaeological and art collections. The prototype described in this paper uses, as study case, 3D models of archaeological objects belonging to Museo del Templo Mayor, a Mexican institution that preserves one of the largest collections of Aztec cultural heritage.

**Conclusions:**

This work is part of an ongoing project focused on creating generic methodologies and user-friendly computational tools for shape analysis for the benefit of scholars and students interested in describing, interpreting and disseminating new knowledge about the morphology of cultural objects.

## Introduction

Around the world, many professionals face the challenge of disseminating information of cultural heritage collections in such a way that objects can be known and studied, anywhere in the world, and preferably without the need of physical contact to guarantee their long-term preservation ( Ekengren *et al.*, 2021). To achieve that goal, cultural institutions have embarked on ambitious 3D digitisation projects and researchers have been looking for better means to improve access to the resulting 3D models ( Clark *et al.*, 2002; Ekengren *et al.,* 2021).

Digitisation indeed have been very successful thanks to the surprising evolution of photogrammetry and laser scanning, which makes possible to model the surface of objects with little effort and in a relatively short period of time ( Pieraccini *et al.,* 2001). Photo-modelling, for example, uses principles of projective geometry to calculate 3D coordinates from overlapping areas of two or more photographs taken from different perspectives. Points representing an object’s feature in one image are matched with homologous points in other images. This allows acquiring the point cloud that represents the surface of the object and at the same time its external appearance (texture). In this way, the technique integrates surveying, modelling and representation into a single workflow ( Bianchini *et al*., 2015). Structure from Motion (SfM), another common technique, also detects matching features from overlapping images to create a point cloud, but as its name suggests, the acquisition of images is done using one or several moving sensors around the target object. While photo-modelling is typically applied to objects, SfM is more common for modelling architectural structures or for creating digital elevation models (DEMs), in which case Unmanned Aerial Vehicles (UAVs) are frequently used for acquiring images from videos. SfM has also been used to digitise historical maps and documents ( Brandolini & Patrucco, 2019).

The continued adoption of these techniques has generated thousands, if not millions, of 3D digital models valuable for research and conservation. The geometric and morphological analysis of such models, for example, is now common in the cultural heritage field, as the bibliographic survey by Pintus *et al.* (2016) demonstrates.

Unfortunately, the search for better means to access collections has not achieved the same level of success. Many times, digital models are produced and then stored in databases without implementing appropriate means to retrieve the 3D information ( Ekengren *et al.*, 2021; Koller *et al.*, 2009). In the case of entity-relationship databases, the simplest way to locate models consists of formulating queries by using keywords that describe the objects’ features. During a search operation, the system is instructed to retrieve all 3D models corresponding, for example, to “tripod vessels” or “anthropomorphic figures”. However, this strategy works only if the categories used in the query coincide with those defined for that particular repository. For instance, if the term *bowl* does not exist in the database thesaurus, the search engine won’t find vessels that are similar but have been registered with another name. Another limiting factor is the language in which the objects are described, because the system might recognize “bowl”, but not *terrine* (French), *cuenco* or *cajete* (Spanish). Additional problems may arise if the most relevant keywords to describe an object are unknown at the time of cataloguing the objects, or if important keywords to identify the objects are unknown to the final users of the system.

Of course, some of these limitations can be overcome by developing multilingual ontologies, an effort that implies agreement, among many expert scholars, on the categories and concepts relevant to describe an object collection ( Almeida & Costa, 2021; Benjamins *et al*., 2004; Uschold & Grüninger, 1996). This has been a research subject in the field of Semantic Web and Linked Open Data. One of the most notable results is the development of CIDOC-CRM -a multilingual description conceptual reference model, which was specifically developed for the sector of galleries, libraries, archives and museums (GLAM). This has become a standard model that many institutions extend and adapt to facilitate the description and exchange of information among their repositories. Within this environment, the exchange of multimedia information is based on the implementation of web service protocols that make contents and semantics of the data sources machine operable and interoperable, though in this regard no standard exists yet ( Bikakis *et al.,* 2021; Crofts *et al*., 2011). A system that uses the CIDOC CRM ontology is SCULPTEUR, developed for searching and retrieving digital images, 3D models, and free text documents using a combination of content-based examples, textual metadata and ontological concepts. Computer query operations are supported by a web service protocol called Z39.50 that allows remote applications to access multimedia data from several museums ( Addis *et al.*, 2003, 2005; Goodall *et al.,* 2004; Sinclair *et al.,* 2024).

These advances have brought benefits such as the possibility to make data interactive, integrated, and contextualized, as well as to record provenance and facilitate logical inferencing, as well as to achieve Web persistence, machine readability, and content repurposing ( Isaksen, 2011:10). Furthermore, semantic web and linked data technologies also allow customizing the retrieval of information based on user profiles and preferences, which results in personalised experiences ( Bikakis *et al.,* 2021).

However, to make these benefits a reality it is also necessary to develop an annotation system that links the ontology to corresponding parts of objects ( Benjamins *et al.,* 2004). This in turns involves implementing segmentation tools for tagging features in images or 3D surfaces that correspond to meaningful elements of cultural heritage entities.

Segmentation and annotation have been major challenges for some time ( Benjamins & Fensel 1998; Hayes-Roth *et al*., 1983) and still are, especially when dealing with 3D models. These tasks can be performed either through manual procedures - a time consuming effort - or with semi-automatic tools ( Grilli & Remondino, 2019). Some successful projects are reported in the field of architecture ( Croce *et al.,* 2021; Grilli *et al.,* 2019; Grilli & Remondino, 2019, Roussel & De Luca, 2023; Teruggi *et al.,* 2020). For example, manual segmentation and annotation through interactive software has been developed for managing thousands of condition reports, architectural descriptions, chemical and physical analysis generated during the restoration of Notre Dame Cathedral ( Roussel & De Luca, 2023). The system operates through a platform called *Äioli* that allows tracing the contour of an architectural feature (column, floor, window, etc.) on an image to establish a link between the 2D and 3D representations of the target section. In this and other applications queries are still based on textual input ( Roussel & De Luca, 2023). Another example is the system developed by Croce *et al*. (2021), which manually label a training set of shape features to then apply a Random Forest Classifier (RFC) to semantically annotate the different parts of a 3D model of the Grand Ducal Cloister in Pisa. Another example using RFC is the multi-level multi-resolution approach to classify 3D point clouds from Milan Cathedral and Pomposa Abbey in Ferrara ( Teruggi *et al*., 2020).

As for semi or automatic segmentation, recent advances in deep-learning methods are promising but no ideal solution exists yet (see a survey of techniques in Attene *et al.*, 2006; Croce *et al.,* 2020; García-García *et al.*, 2017; Grilli *et al.*, 2017; Shamir, 2006). Deep-learning algorithms, however, focused on segmenting objects according to their geometric properties, regardless if these have a semantic meaning for the final user ( Attene *et al.,* 2009). Cultural heritage objects are particularly challenging for this kind of approaches, because contrary to architecture in which parts of a building (columns, porticos, doors, windows etc.) have certain standard structures, artefacts y objects in general present an enormous diversity of features, which complicates the recognition task. Also, the techniques have to account for the fuzziness in the boundaries of those features. As Attene *et al*. (2009) point out: “… in a human body model the neck may be considered part of both the head and the torso”. To complicate matters further, the training of deep-learning models relies on the availability of massive amounts of data, a condition rarely met in cultural heritage applications, though this limitation can be reduced with transfer-learning, an approach in which the recognition capabilities of a model trained with massive generic data are extrapolated to analyse a smaller sample of data.

But whatever the means to describe collections and share information, it would be appropriate to have a system that analyses the intrinsic visual characteristics of the objects, specifically a system that process queries recognising the shape of objects without relying exclusively on keywords as search criteria ( *i.e.* Content-based Information Retrieval or CBIR). One step for the development of such a system is computing a numerical representations of shape for each object ( *i.e.* its shape descriptor), and the implementation of algorithms that compare all the shape descriptors stored in a database ( *i.e.* a matching operation) to facilitate the retrieval of 3D models ( Funkhouser *et al.,* 2003; Tangelder & Veltkamp, 2008).

This paper describes the first stage of an ongoing project oriented to such goal. It describes the implementation of the first module (MeshAnalizer) of a system called ArcheoShape, based on three types of shape descriptors and four dissimilarity measures that facilitate the matching and retrieval operations. The module is called MeshAnalizer and will function as a kind of search engine that instead of using keywords will recognize objects automatically by comparing their numerical shape descriptions. Future work will integrate deep-learning techniques for annotation and segmentation. The benefits of this system are conspicuous in the context of museums, where it is necessary to find and retrieve 3D models from large collections.

### Basic requirements

**Figure 1.**
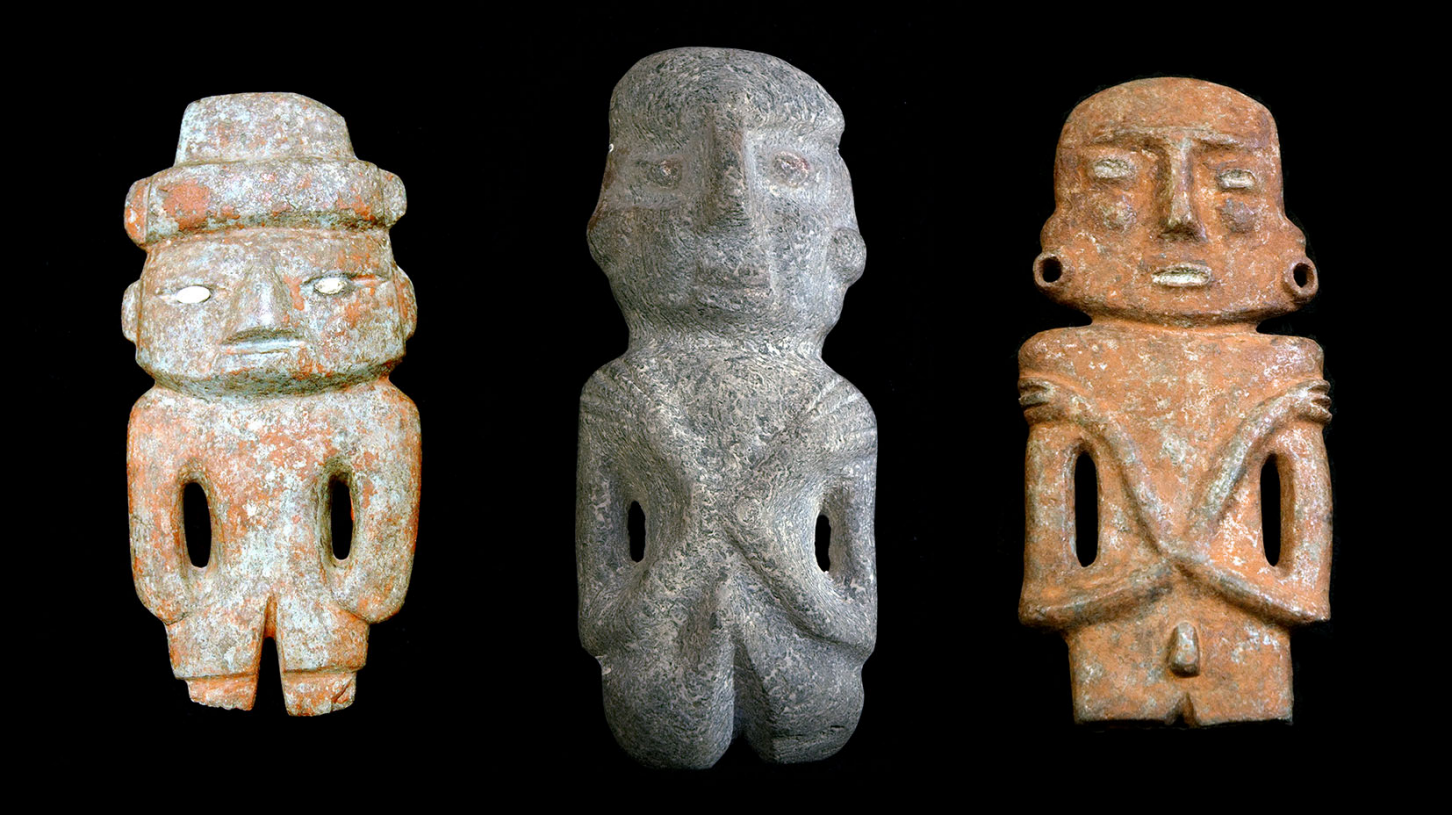
Three figurines found in the Sacred Precinct of Tenochtitlan. They are similar in their overall geometry, but differ in some details, such as the design of the head and the position of the arms. A recognition system must be able to find the 3D models of these objects, despite their partial differences in shape.

A system of shape recognition must be able to discover shape similarities between partially isometric objects, that is, between objects that share shape characteristics even if they are not identical. For example, a researcher might need 3D models of all the anthropomorphic figurines in a museum. In this case, the query should not be affected by the fact that one object lacks a head, while another is missing a leg. Also, it must retrieve several complete models of the same class, regardless of whether they differ in certain details ( Gal & Cohen-Or, 2006). The three objects shown in Figure 1 illustrate this situation; they belong to the same class of anthropomorphic figures, but their heads and arms show some morphological differences.

The second requirement is that the system be able to detect similarities without being affected by affine variations such as rotation, translation, reflection and scale of the 3D models. Ideally, different combinations of translation, rotation, and scale applied to equal objects during the digitisation process should not affect the system’s capacity to recognise their similarity.

To fulfil those requirements, it is necessary to apply a specialized set of methods of computer vision specifically designed to identify objects’ similarities efficiently, within the processing limits of today’s computers and that are sufficiently discriminatory to resolve the requirements of cultural heritage institutions.

### Development of a search-engine module

The development of shape recognition systems has been the subject of research since the 1980s, especially in the fields of computer vision, geometric modelling, and machine learning ( Besl & Jain, 1985; Bustos *et al.*, 2004, 2005; Campbell & Flynn 2001; Jain & Mishra 2014; Lara López *et al*., 2017; Loncaric 1998; Tangelder & Veltkamp 2008; Theologou *et al.*, 2014; Veltkamp & Hagedoorn, 1999). In the early 2000’s. Funkhouser *et al*. (2003) developed a system for retrieving 3D models applying the shape descriptor of spherical harmonics, implementing an interface that allowed queries by example and also sketch-based searches. Another early system was developed by Paquet and Rioux (2000) with shape descriptors based on tensors of inertia, distribution of normal vectors, distribution of cords and multiresolution analysis. Its interface allows entering a combination of parameters such as scale, shape or colour to search the database. Other innovations were due to Suzuki (2001) who included the processing of material data (colour and texture) as search criteria; although its descriptors require normalization of 3D models *via* PCA.

In the field of cultural heritage, Rowe *et al.* (2001) and Rowe and Razdan (2002) implemented a shape-based search engine for analysis and retrieval of native American ceramic vessels. Objects were modelled as parametric surfaces and the interface allows query by example and sketch-based query, and links to descriptive data. Another interesting system was designed by Schurmans *et al*. (2002) according to specific archaeological research objectives. For example, indicators of craft specialization can be gathered from the morphology of ceramic vessels. This involves matching shapes, as well as text, numeric, and vessel data calculated with the system tools.

Those projects demonstrate that the effectiveness of a recognition system depends above all on implementing efficiently two basic procedures. The first one consists in calculating a “shape descriptor”, that is a numerical representation of the form of each 3D model. Such descriptor can represent the global geometry of the object or a sample of its local features. The computation of shape descriptors involves a combination of mathematical, statistical, and more recently Machine Learning methods to represent shape in a numerical array or feature vector ( Tangelder & Veltkamp, 2008). Some examples of characteristics encoded by shape descriptors are the curvature or orientation of a certain quantity of patches drawn around points chosen in a random manner ( Jiménez-Badillo *et al.,* 2013), or alternately, signals calculated with spherical functions ( Kazhdan & Funkhouser, 2002; Kazhdan *et al.* 2003), reflective symmetry ( Kazhdan *et al.*, 2002, 2004a, 2004b), spin-images ( Johnson and Hebert, 1999), shape-contexts ( Mori *et al.,* 2001), histograms of spherical orientations ( Roman-Rangel *et al.*, 2016), and many others as described in bibliographic surveys by Bustos *et al.* (2004, 2005), Lara López *et al*. (2017), and Rostami *et al.* (2019).

More recently, deep-learning models have been developed to compute shape descriptors for the recognition of generic objects. The spectrum of techniques includes supervised and self-supervised approaches ( Herrewegen *et al.,* 2023), such as Neural Networks ( Krestenitis *et al.,* 2020; Xie *et al.,* 2017; Yang *et al.*, 2021), Convolutional Neural Networks ( Feng *et al.*, 2018; Kim & Chae, 2022), Autoencoders ( Furuya & Ohbuchi, 2020), Point Cloud Networks ( Wang *et al.*, 2020), Graph Neural Networks ( He *et al.*, 2020), and Geometric Deep Learning techniques ( Bronstein *et al*., 2017). These methods, however, require large data sets for training the models, a condition rarely met by cultural heritage collections, including the one used for this project.

In any case, the final objective is that the shape of the object is characterized in the best possible manner, as to constitute a “signature” (numerical representation) of the object readable by a computer. As mentioned above, the numerical descriptor must represent the shape regardless the object’s position, orientation and scale.

The second procedure consists in creating an index of the numerical representations of all the objects ( *i.e.* their shape descriptors), to facilitate the matching operation. The comparison between 3D models is done by measuring their degree of similarity with a mathematical function, such as Euclidean distance, that indicates the degree of their resemblance, so that when a query is implemented the system can rank objects from the more to the less similar ( Bustos *et al.,* 2004; Tangelder & Veltkamp, 2008).

The search-engine module developed over the course of this project is based on the implementation of three different global descriptors, namely shape distributions ( Osada *et al.,* 2002), reflective symmetry ( Kazhdan *et al.*, 2002, 2004a, 2004b) and spherical harmonic functions ( Kazhdan and Funkhouser, 2002; Kazhdan *et al.,* 2003).

As for determining the degree of dissimilarity between objects, four measures have been implemented: Euclidean distance, City block (Manhattan) distance, Chebychev distance, ^1^ and Minimum Coordinate distance ( Table 1). In the case of shape distributions, each dissimilarity measure has been implemented in two norms: the probability density function (pdf), and the cumulative distribution function.

**Table 1.** The four distance measures implemented in the prototype to calculate dissimilarity of shape descriptors.

Dissimilarity measure	Definition
**Euclidian distance**	$\sqrt{\sum_{i=1}^{n}{\left(x_{i}-y_{i}\right)}^{2}}$
**“City block” distance**	$\sum_{i=1}^{n}\left|x_{i}-y_{i}\right|$
**Chebychev distance**	$\max\left(\left|x_{i}-y_{i}\right|\right)$
**Minimum coordinate distance**	$\min\left(\left|x_{i}-y_{i}\right|\right)$

The following sections describe, in layman’s terms, the methods to compute the three shape descriptors selected for the implementation of the search-engine module.

### Shape distributions

The simplest shape descriptors included in the search-engine module are five probability distributions proposed by Osada *et al.* (2002). The names given to the descriptors depend on the type of calculation, “A” stands for angle and “D” for distance:

• A3: The angle between three random points on the surface of the 3D model.
• D1: The distance between the centroid of the model and one random point on its surface.
• D2: The distance between two random points on the surface.
• D3: The square root of the area of the triangle formed by three random points sampled on the surface.
• D4: The cube root of the volume of the tetrahedron formed by four random points sampled on the surface.

As Osada *et al.* (2002) recommend, we compute those variables for a very large sample of points selected from the surface-mesh of each 3D model, specifically 1,048,576 points ( *i.e.* 1024 × 1024 points). The measurements were then transformed into a frequency histogram (probability distribution), which could then be used as the global signature of the object’s shape. Once the shape histograms for all the objects had been computed, a normalization step was necessary to standardize the scales of all the histograms in order to avoid matching errors due to variations in the size of the objects. The objective is finding the scale that produces the minimal dissimilarity measure during the comparison of two object’s histograms. To achieve this, one of the methods proposed by Osada *et al*. (2002) involves the following steps: align both shape distributions ( *i.e.* histograms) so that the mean sample in each distribution equals 1; then find the minimum value *D*( *f* ( *x*), *sg* ( *sx*)) for values of log *s* from -10, 10, in 100 equally spaced intervals; where *f* and *g* represent the shape distributions of two models; x correspond to the 3D model; and D corresponds to the distance function chose from A3, D1, D2, D3, or D4. Finally, select the minimum value among the results and use it as the dissimilarity measure for the two normalised shape distributions. This guarantees that two objects of the same shape but different sizes are recognized as similar, and vice versa, two objects of the same size but different shape are recognised as different.

The resemblance between any pair of objects can be determined by applying a function that measures dissimilarity between distributions ( *i.e.* histograms), for example Euclidean distance or any of the other measures mentioned in Table 1.

**Figure 2.**
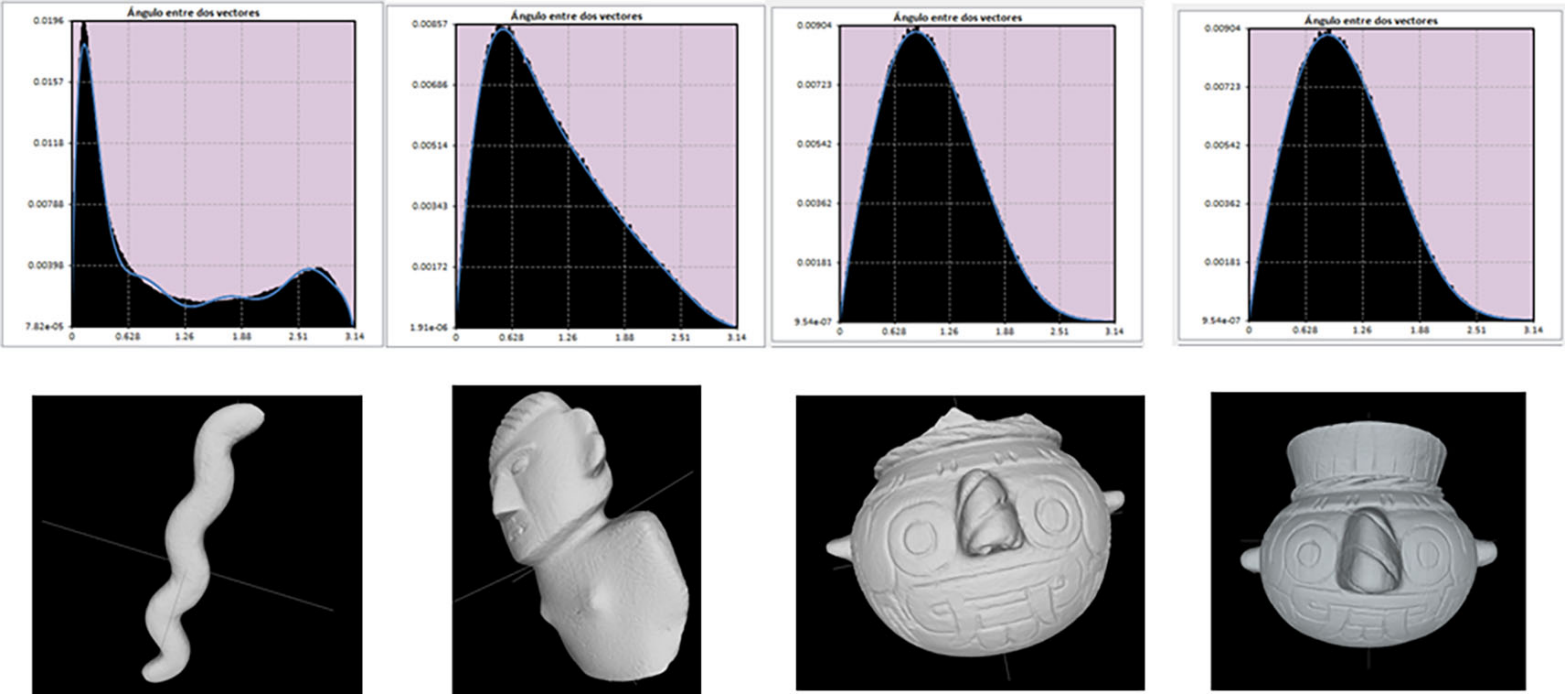
Frequency probability histograms resulting from obtaining measurements of angles between vectors for four objects from the Templo Mayor museum collection. Notice the great difference between the histogram of the serpentiform scepter (first object on the left), which shows the mode close to zero, and the histogram of the flat anthropomorphic figure (second from left to right). On the other hand, the great similarity of the histograms corresponding to the so-called Tlaloc vessels located on the right side can be appreciated. Comparing these histograms allows the recognition system to determine whether or not two objects belong to the same class.

Figure 2 shows histograms resulting from the descriptor A3 (angle between three random points), representing the shape of four archaeological objects. Notice the probability distributions of the two models on the left, reflecting the differences between the long, wavy form of the serpentiform sceptre and the flat, wide anthropomorphic figurine. For an elongated figure, the angles between vectors tend to concentrate around the mode, while for flat objects the histogram would have a flat distribution because there would not be a predominant value of angle between vectors. In contrast, the images on the right correspond to two vessels whose histograms are quite similar because their shapes are also alike. Through this kind of comparison, the recognition system manages to identify similarities or differences between archaeological objects.

### Reflective symmetry descriptor

The second representation of shape, more complex but at the same time more effective, is the reflective symmetry descriptor proposed by Kazhdan et al. (2002, 2004a, 2004b). As these authors point out, symmetry —or the lack of it — is one of the most distinctive characteristics of any object.

Given a 3D model, denoted by function *g*, the concept of reflective symmetry implies that there is a reflection function *γ*, such that $g=\gamma \left(g\right)$ . This means that the pointwise distance between the points of surface *g* and the points of surface $\gamma \left(g\right)$ is zero *.*

Kazhdan *et al.* (2002) propose quantifying reflective symmetry with respect to several cutting planes, oriented on perpendicular axes that pass through the centroid of the model. For any given plane *P* cutting the shape *f*, the method consists in finding the function *g* such that g=γ with $⟦f-g⟧$ as small as possible *.* Mathematically this is expressed as:

$$\mathrm{SD}\left(f,\gamma \right)=\min_{g|\gamma \left(g\right)=g}⟦f-g⟧$$ (1)

where SD stands for Symmetry Descriptor. The more symmetric the shape *f* with respect to plane *P*, the smaller the value of $\left\|f-g\right\|$ *.* Large values of $\left\|f-g\right\|$ indicate that the surface is less symmetric.

**Figure 3.**
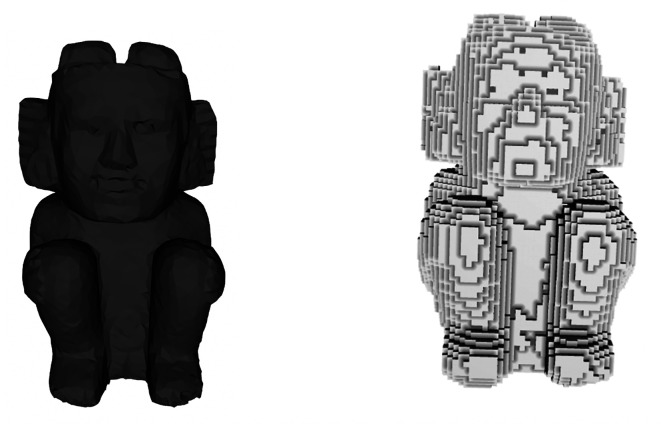
A 3D model of an archaeological figure and its voxel representation.

The calculation of reflective symmetry can be performed quicker and more efficiently by transforming the description of the surface mesh (3D model) into a discrete volumetric representation ( *i.e.* voxel grid). The process starts by immersing the triangular surface mesh inside a regular 3D grid. When a triangle of the mesh intersects a voxel of the 3D grid, such voxel is assigned a value of 1. Such rasterization process allows determining where the points and triangles of the mesh are located on the 3D grid. Notice that there are voxels in the grid that do not intersect the 3D mesh and therefore lack any information. For calculating the reflective symmetry descriptor, it is convenient to add to these voxels information related to how far they are from the surface of the model, for which the distance transform is used. This transform consists of assigning each voxel the distance to the nearest voxel belonging to the model. Then, the distance is transformed to a measure of similarity with the Gaussian function. Additional voxelization methods available can be found in Aleksandrov *et al*. (2021) and Huang *et al.* (1998). The resulting discrete representation consists of a 3D set of voxels, which appears like the archaeological model shown in Figure 3. ^2^

Once the voxel grid has been labeled in this way, the descriptor can be calculated. Broadly speaking, what it is done is to assume that when passing a cutting plane through the voxel grid there is perfect reflective symmetry between the two halves, so that one of the two halves can be replaced with the other if that property were fulfilled. Then, the reflective symmetry distance is calculated in the real model and compare it with the assumed model to measure any difference. If both representations are equal (zero distance), then there is perfect symmetry and a radius of 1 is assigned to the corresponding plane. If not, a value less than 1 is assigned according to how different these figures are.

**Figure 4.**
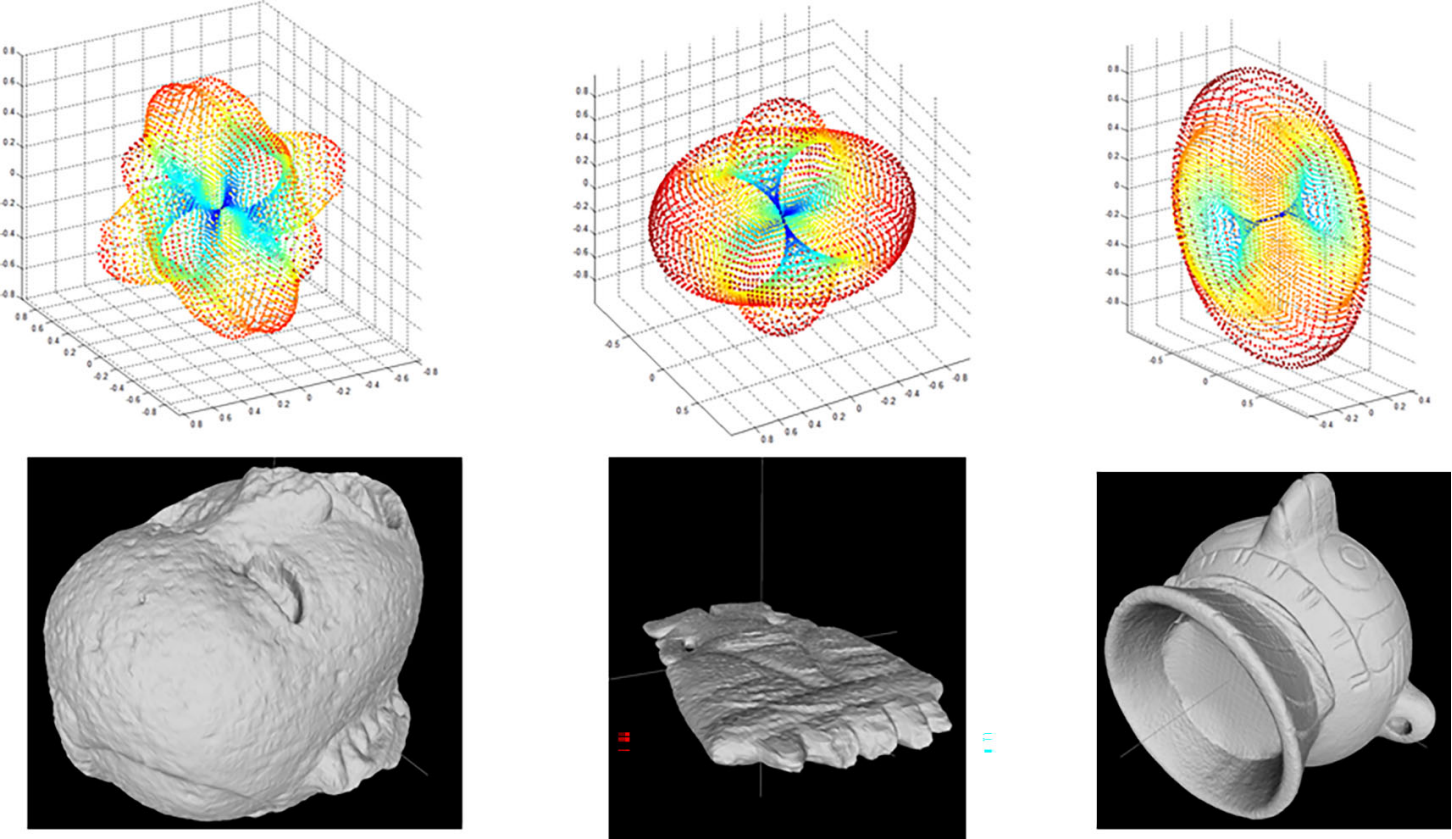
Graphs of the reflexive symmetry descriptors obtained for three objects from the Templo Mayor collection.

Finally, the measures of symmetry obtained from a number of cutting planes ( *i.e.* axes of symmetry) are concatenated to generate a 3D graph, describing the model’s global symmetries. Values near to 1 indicate perfect symmetry, while those near zero indicate that the two halves of a model are too asymmetrical. This graph is used to compare an object with any other for the matching and retrieval application. Visual representations of the descriptors obtained from three archaeological models are shown in Figure 4.

As with the histograms shown in Figure 2, reflexive symmetry descriptors offer another method for calculating dissimilarity between 3D models.

### Spherical harmonics descriptor

A third way of describing shapes on a computer is to consider them as outcomes of mathematical functions. Each stroke of a drawing, for example, can be regarded as a mix of 2D functions. The numerical representation of the whole drawing would be the sum of many functions. In practice, the functions are unknown, but they can be calculated by applying standard mathematical procedures such as the Fourier Transform, which would find the specific mix of simple functions that represent the complete drawing.

**Figure 5.**
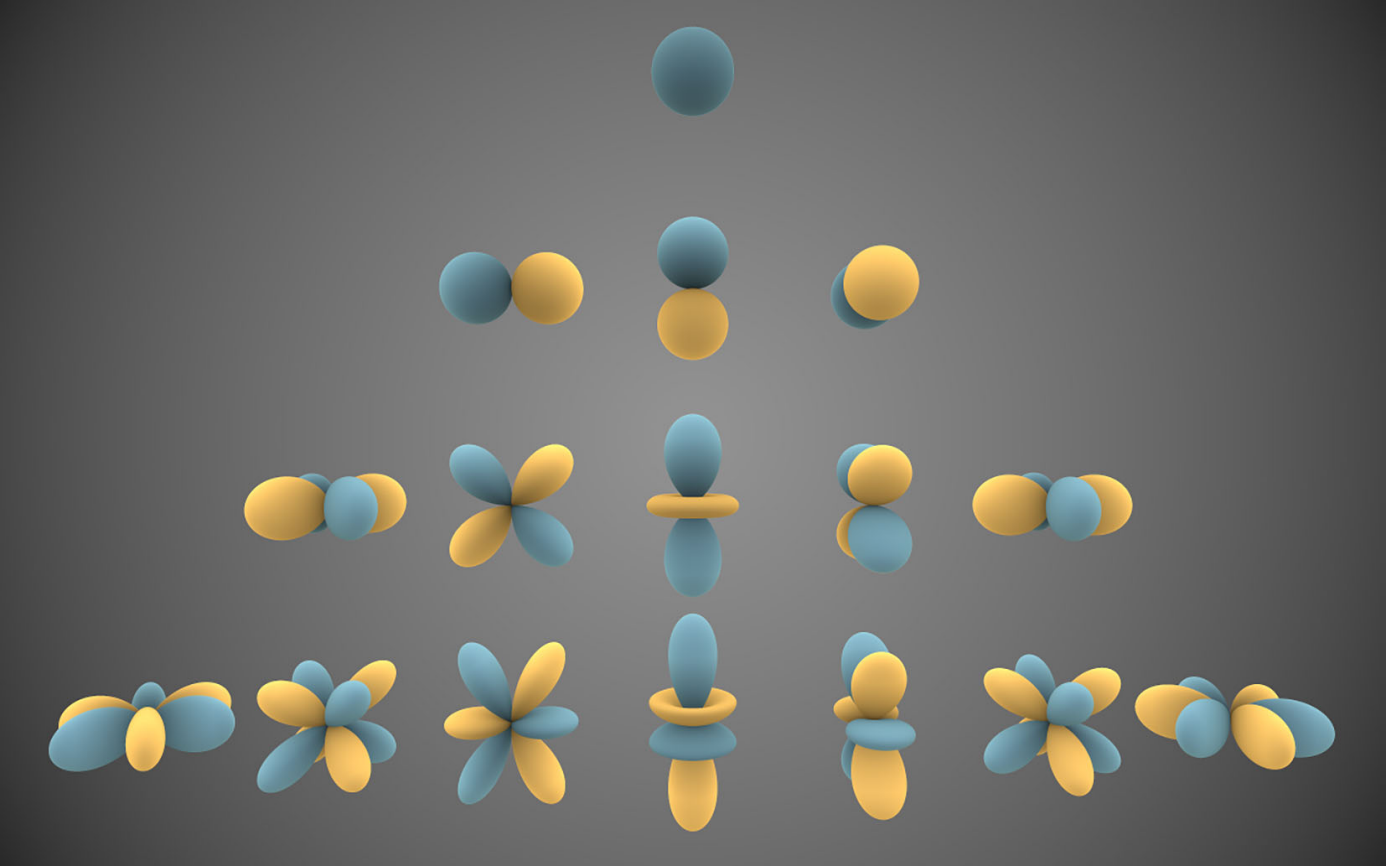
Graphical representation of harmonic functions calculated to obtain shape descriptors from 3D models.

Something similar happens with 3D objects, but in that case the function describing the shape is defined on the surface of the sphere. One way of describing the shape of a surface is calculating the so-called spherical harmonic functions ( Figure 5). Intuitively, we can think of the spherical harmonic functions as “Lego” pieces that, together, help built the shape of complex 3D objects. This is possible, because mathematically speaking, spherical harmonics constitute a complete set of orthogonal functions and therefore form an orthonormal basis, upon which any function defined on the sphere (like the shape of a 3D model) can be expressed as the sum of these spherical harmonics.

The simplest function is the sphere and from there higher order functions are derived. To obtain the mathematical description of a 3D model it is necessary to decompose the general form into constituent elements. The more complex the shape of an object, the more harmonic functions will be needed to describe it. The results of the decomposition are used to compare the shape of 3D models. Image by Inigo.quilez - Own work, CC BY-SA 3.0, (available here).

The exact combination of spherical harmonic functions needed to describe a particular object can be found though a harmonic analysis. This method divides the complex surface of a 3D model into sums of relatively simple components.

Harmonic analysis is the branch of mathematics that deals with the problem of representing functions as the combination of basic elements called waves or harmonics. Typically, the term harmonic refers to functions with sinusoidal variations, but more strictly, it indicates any solution of Laplace’s Equation. The Fourier series is an example of a complete set of harmonics, which consists of sine and cosine waves of different frequencies.

In this work, we adopted the Spherical Harmonic Transform (SHT) to obtain a reduced representation of a 3D mesh. This method is a powerful tool for describing data on a sphere using spherical harmonics as basic functions. Given a function $f\left(\theta ,\varphi \right)$ in the spherical coordinates θ and φ the decomposition of $f\left(\theta ,\varphi \right)$ in spherical harmonics $Y_{l}^{m}\left(\theta ,\varphi \right)$ is written as:

$$f\left(\theta ,\varphi \right)=\sum_{l=0}^{\infty }\sum_{\left|m\right|\leq l}c_{\mathrm{lm}}Y_{l}^{m}\left(\theta ,\varphi \right).$$ (2)

Here, l ≥ 0 and *m* are integers such that $\left|m\right|\leq l$ , $c_{\mathrm{lm}}$ is the coefficient of the harmonic $Y_{l}^{m}\left(\theta ,\varphi \right)$ , and the general form of $Y_{l}^{m}\left(\theta ,\varphi \right)$ is:

$$Y_{l}^{m}\left(\theta ,\varphi \right)=\sqrt{\frac{2l+1}{4\pi }\frac{\left(l-m\right)!}{\left(l+m\right)!}}P_{l}^{m}\left(\cos\theta \right)e^{\mathrm{im\varphi}},$$ (3)

where $P_{l}^{m}\left(x\right)$ is a Legendre polynomial:

$$P_{l}^{m}\left(x\right)=\frac{{\left(-1\right)}^{m}}{2^{l}l!}{\left(1-x^{2}\right)}^{\frac{m}{2}}\frac{d^{l+m}}{{\mathrm{dx}}^{l+m}}{\left(x^{2}-1\right)}^{l}.$$ (4)

The problem in the Spherical Harmonic Transform is to calculate the coefficients $c_{\mathrm{lm}}$ .

In practice, it is not possible to calculate the coefficients of all the spherical harmonic functions. For this reason, we limit the order of the harmonics to a fixed value b (for instance 16 or 32) so that:

$$f\left(\theta ,\varphi \right)\approx \sum_{l=0}^{b}\sum_{\left|m\right|\leq l}c_{\mathrm{lm}}Y_{l}^{m}\left(\theta ,\varphi \right).$$ (5)

Finally, the coefficients $c_{\mathrm{lm}}$ are estimated by finding the least-squares solution to equation (5). That is to say, for a set of *n* points $\left\{\left(\theta_{1},\varphi_{1}\right),\left(\theta_{2},\varphi_{2}\right),\ldots ,\left(\theta_{n},\varphi_{n}\right)\right\}$ where $f\left(\theta ,\varphi \right)$ is evaluated, we calculate the values of the coefficients $c_{\mathrm{lm}}$ that minimize:

$$\sum_{i=1}^{n}{\left(f\left(\theta_{i},\varphi_{i}\right)-\sum_{l=0}^{b}\sum_{\left|m\right|\leq l}c_{\mathrm{lm}}Y_{l}^{m}\left(\theta_{i},\varphi_{i}\right)\right)}^{2}$$ (6)

To describe a 3D mesh using the Spherical Harmonic Transform, we define the function $f^{r}\left(\theta ,\varphi \right)$ as the intersection between the voxelized version of the 3D mesh and the sphere of radix *r*, both centered at the origin. The function $f^{r}\left(\theta ,\varphi \right)$ takes the value 1 only if the sphere intersects a voxel of the mesh at the point (θ,φ ), otherwise, this function is 0. The Spherical Harmonic Transform is applied to different radii so that the functions $f^{r}\left(\theta ,\varphi \right)$ are characterized by their corresponding harmonic coefficients.

The simplest harmonic function is the sphere, so if the object resembles a balloon, only one harmonic component of degree zero is enough for describing it. However, if the model has a more complex shape, then it is essential to calculate several higher order harmonic functions.

**Figure 6.**
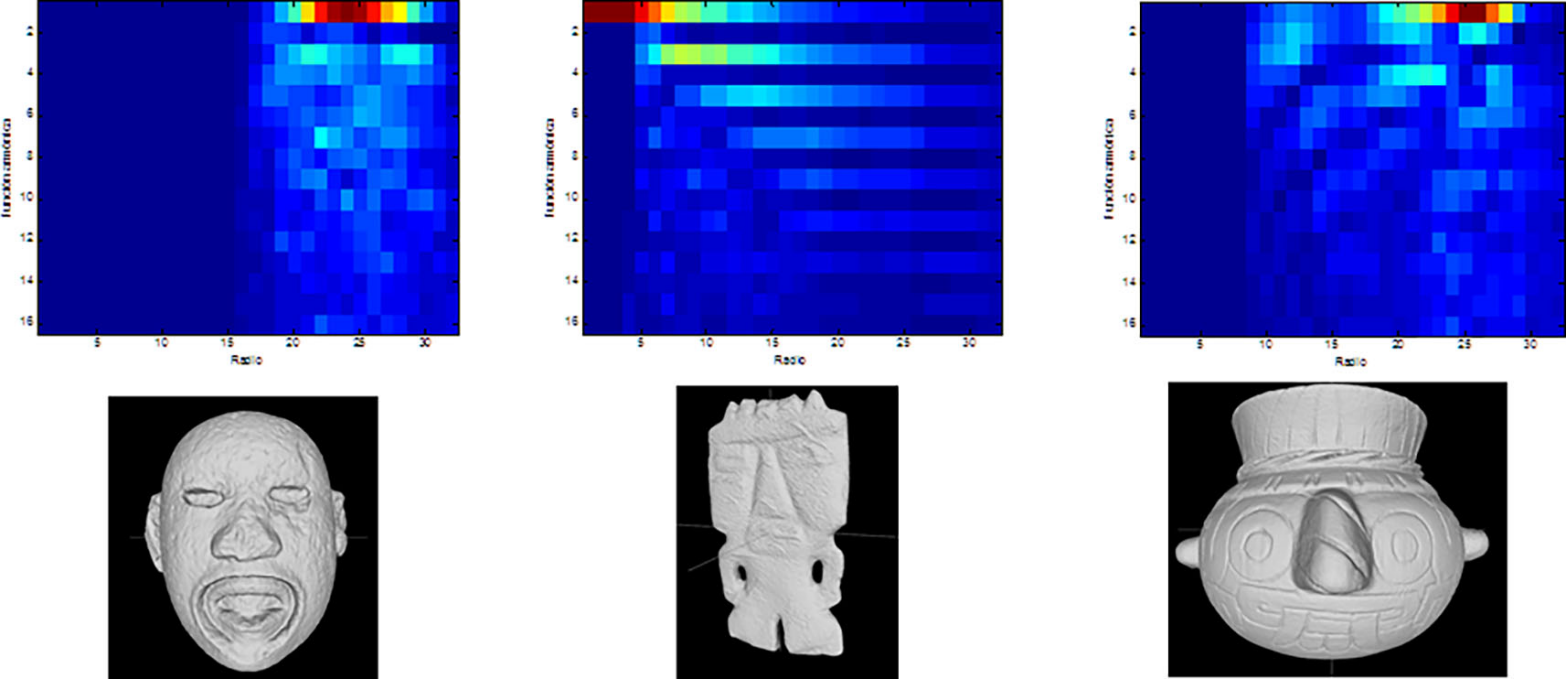
Graphs of the decomposition coefficients obtained by calculating harmonic functions for three objects of the collection of the Templo Mayor collection.

There are several methods to compute shape descriptors based on spherical harmonics. Some require a priori registration of the model along principal axes ( Saupe and Vranic 2001; Vranic & Saupe, 2001; Vranic *et al.,* 2001), but these are not good to process 3D models of the same class digitised with different orientations ( Funkhouser *et al.,* 2003). A method that solves that limitation is the one proposed by Kazhdan and Funkhouser (2002), and Kazhdan *et al.* (2003) and it is the one implemented during this project. In practice, the descriptor is computed as follows:

1. The 3D model is subjected to a voxelization process, like the one applied in the case of reflective symmetry (c.f. Huang *et al.* 1998). The size of the voxel grid is 64 × 64 × 64.
2. The 3D model is aligned with its voxel representation in such a way that is centre of mass coincides with the centre of the voxel grid.
3. A voxel is assigned a value of 1 if it contains any point on the surface of the 3D model, and 0 otherwise.
4. The voxel grid is decomposed into 32 spheres of radii 1 to 32, which produces 32 spherical functions.
5. Each sphere is decomposed as a sum of its first 16 spherical harmonics.
6. Finally, these different signatures are combined to obtain a 32 × 16 signature for the 3D model. The result is a 2D image that represents the decomposition coefficients for each harmonic function and each radii ( Figure 6).

To compare two objects using their harmonic representations, it is simply necessary to compute the Euclidean distance between them: “Thus, finding the K closest models to a query is equivalent to solving the nearest-neighbour problem” ( Kazhdan & Funkhouser, 2002). Figure 6 shows the spherical harmonics descriptor for three objects.

### User interface

Some early systems were tested with collections of 3D models produced with computer-aided design (parametric models) software and acquired on the internet. In contrast, we have developed a first module (MeshAnalyser) of a search engine called ArcheoShape that uses real archaeological objects. At this stage, the objective is to assess how good are the shape descriptors described in the previous sections to match and retrieve real archaeological objects before continuing the development of the entire system and deploying it within a museum environment. The module developed over the course of this project is freely available in the following GitHub repository ( Mendoza-Montoya, 2023b), which contains the source code, written in C++, ready to be compiled in Windows and Linux, as well as an executable file. Instructions to compile are included in the GitHub repository. A sample of ten 3D models of archaeological artefacts are also provided under the Creative Commons Attribution-NonCommercial-NoDerivatives 4.0 International. These resources will allow any user to test the MeshAnalyser module in a local computer, as well as replicate or customized the module for his own purposes.

To test the implementation of the shape descriptors, we used a sample of nearly 500 archaeological artefacts from the Museo del Templo Mayor. The collection is available for research purposes through specific agreements with Instituto Nacional de Antropología e Historia. ^3^

The Museo del Templo Mayor preserves objects discovered between 1978 and 1982 within the area occupied by the Sacred Precinct of Tenochtitlan, the most important religious centre of the Aztecs and nowadays a famous archaeological site adjacent to Zócalo square in Mexico City ( Matos Moctezuma, 1988). The core collection includes more than 8000 objects from ritual offerings found in the main pyramid temple ( *i.e.* Templo Mayor) of the site and its surroundings, and include ritual artefacts, flora, fauna, and human remains ( López Luján, 1994; Nagao, 1985). The collection has increased considerably in recent years thanks to the excavations conducted in the same site by different research teams led by archaeologists Barrera Rivera & Islas Domínguez (2018), Barrera Rodríguez, and López Luján (2012, ), 2019. Indeed, between 2012 and 2019, 43 new offerings, containing 13,925 artefacts and 35,648 samples of organic material have been reported. Digitization of this collection is still at a very early stage, but we have been able to acquire a sample of 495 objects, including stone-masks, anthropomorphic and zoomorphic figures, clay vessels (bowls, pots, jars, braziers), religious paraphernalia like sceptres, earplugs, ritual pendants, as well as flint sacrificial knifes, flutes, and models of drums made of clay or stone.

**Figure 7.**
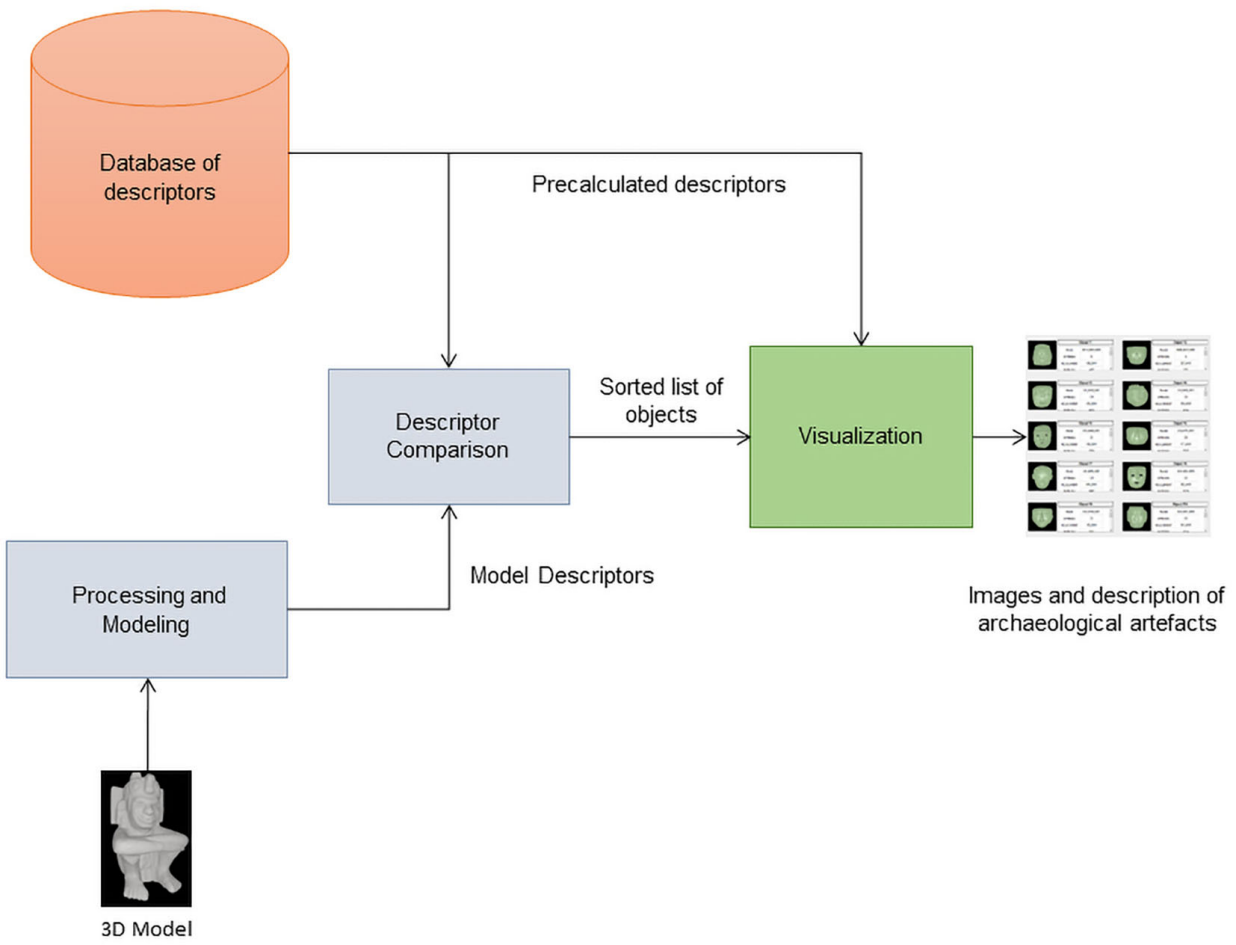
Architecture of MeshAnalyser.

The implementation of the prototype was divided into two independent offline and online jobs. The main offline task consists in computing the shape distributions, reflective symmetry and spherical harmonics descriptors of all the archaeological 3D models available (“Database of descriptors” in Figure 7). The actual matching and retrieval operations are done online.

The module performs four basic operations as follows:

• Mesh processing and modeling. Each mesh entered by the user is down-sampled to subsequently obtain its shape and reflective symmetry descriptors. In the case of the reflective symmetry and spherical harmonics descriptors, the module also performs a previous step consisting in computing a voxelized representation of the mesh.
• Descriptor database management. The system has a collection of descriptors that up to now correspond to around 500 3D models of archaeological artefacts. This process is done offline. The resulting repository is the source against which any query object is compared during a search and retrieval operation and therefore underpins the artefact-matching framework.
• Descriptor comparison. At the core of the system lies the comparison of the query-model input by the final user against the corpus of descriptors stored the internal database. This process yields a list of objects ordered according to the similarity they have with the newly analyzed 3D mesh.
• Results visualization. The interface presents the outcome of the comparative analysis, showcasing images of the archaeological artifacts arrayed in the order determined by the descriptor-matching procedure. This sequential display facilitates an intuitive assessment of similarity.

The system’s architecture is crafted to reflect the sequential execution of these operations ( Figure 7). As mentioned above, we have made an executable file of the module available through the link provided below in the section Software Availability, as well as the source code. Every section of the source code includes detailed comments that will be updated to reflect changes in its functionality. This would facilitate the replication of the software by others as the software evolves.

Next, we explain how these operations interact when a new 3D mesh is introduced into the system.

**Figure 8.**
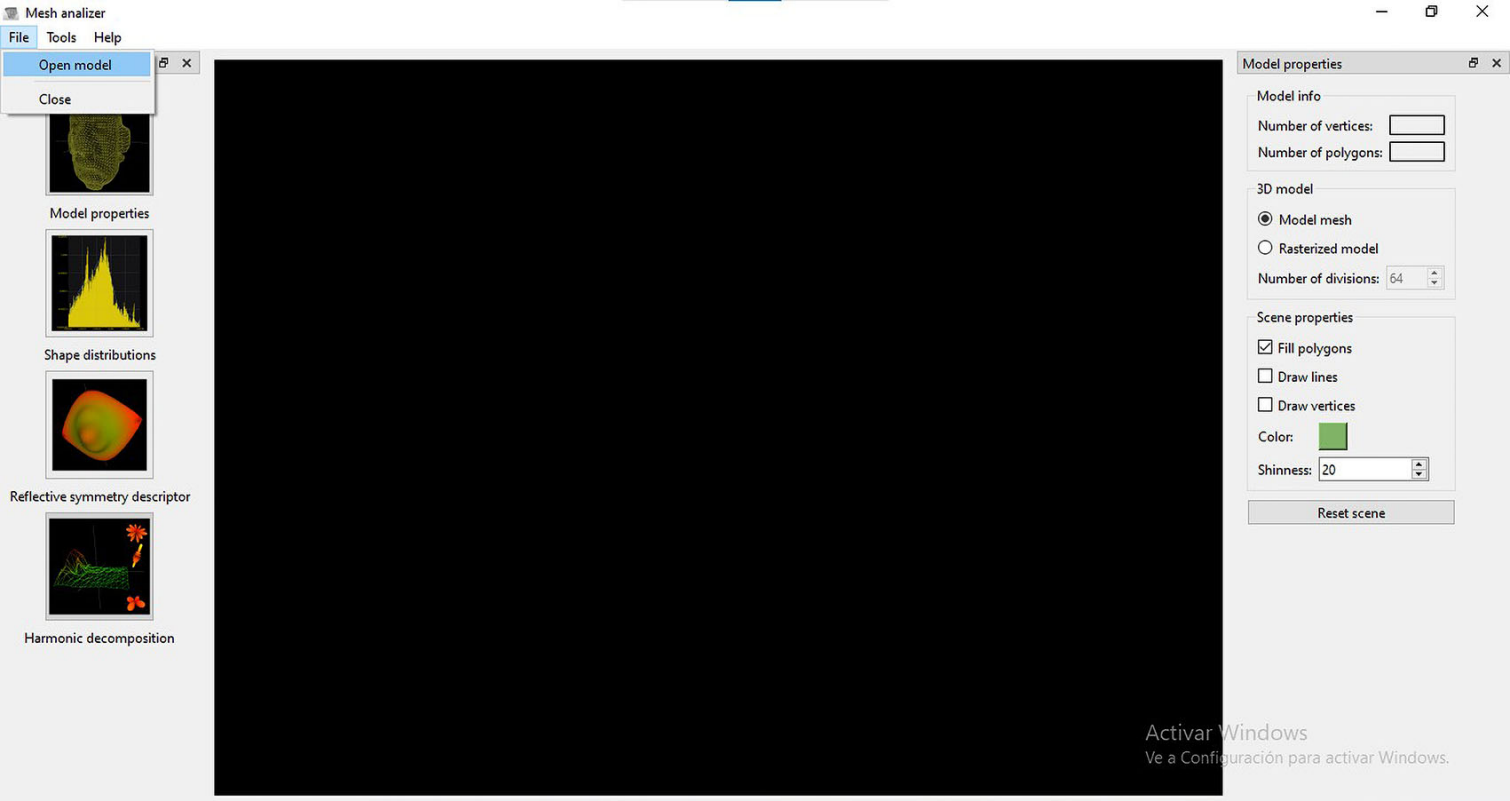
Screenshot of the window to open a file in MeshAnalizer.

**Figure 9.**
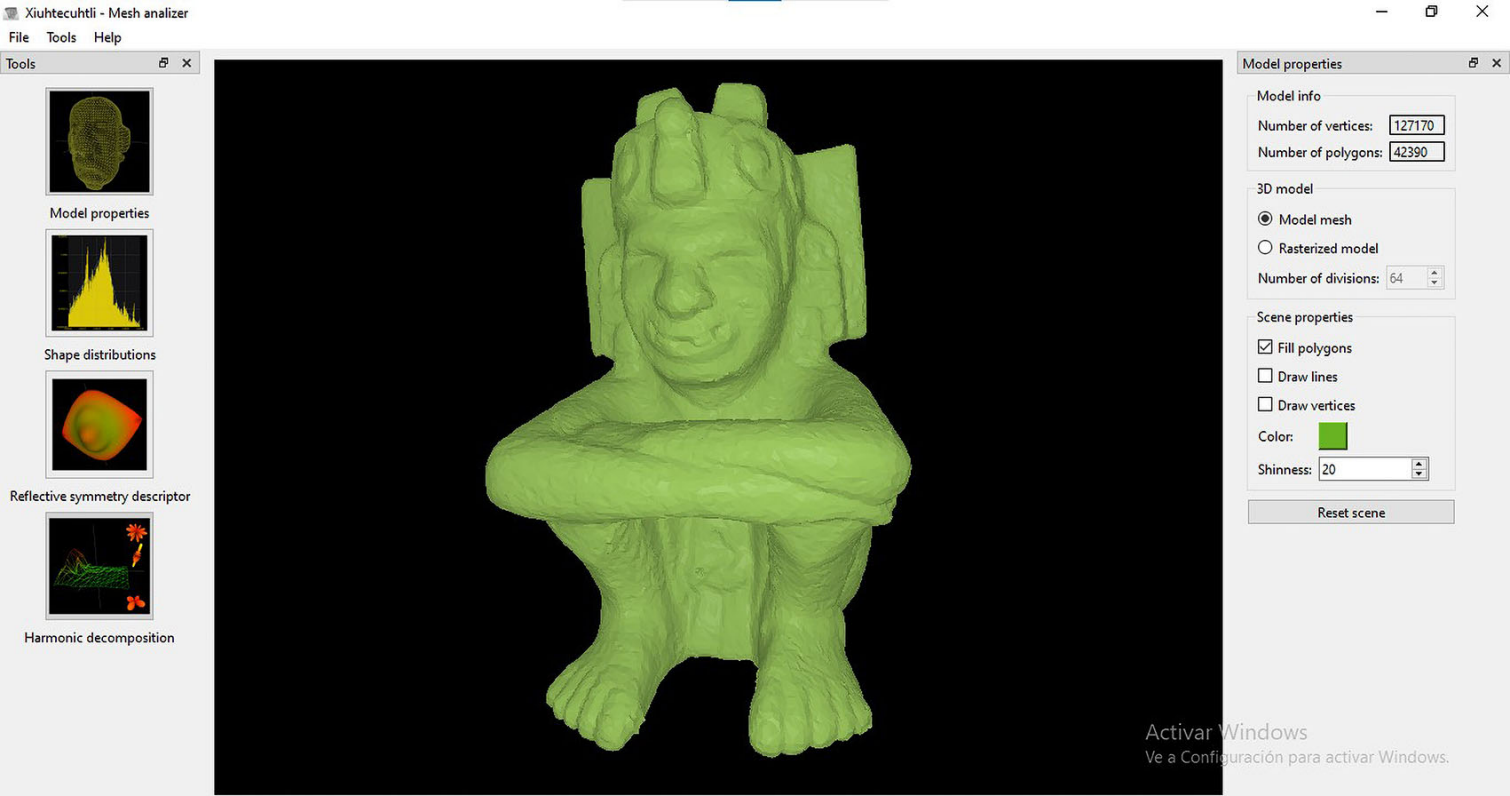
Screenshot of the window that displays the properties of a 3D mesh opened with MeshAnalizer.

**Figure 10.**
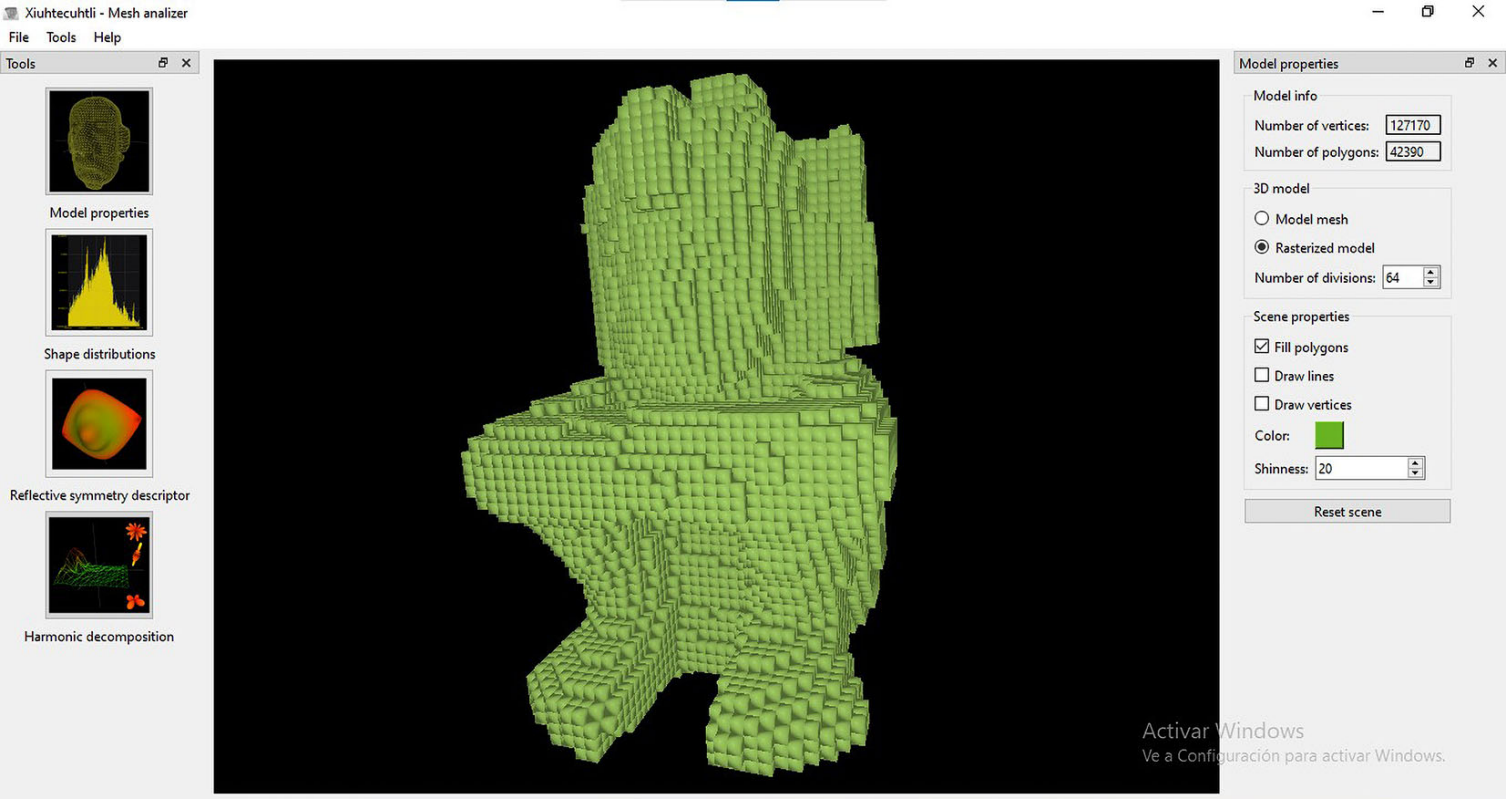
Rendering a triangular mesh as a voxelised model.

**Figure 11.**
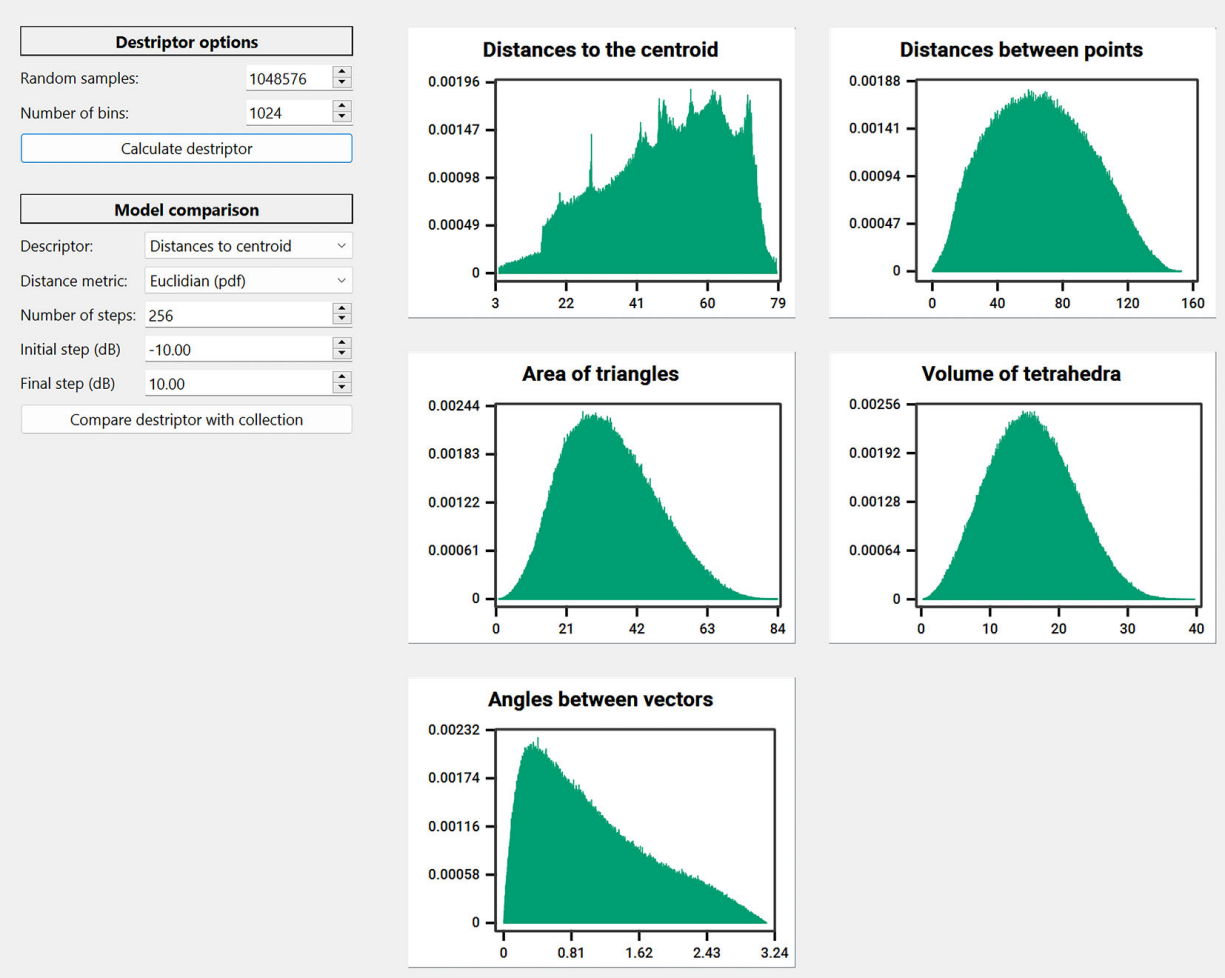
Screenshot of the window to apply the Shape Distribution algorithm to perform a matching and retrieval operation with MeshAnalizer.

**Figure 12.**
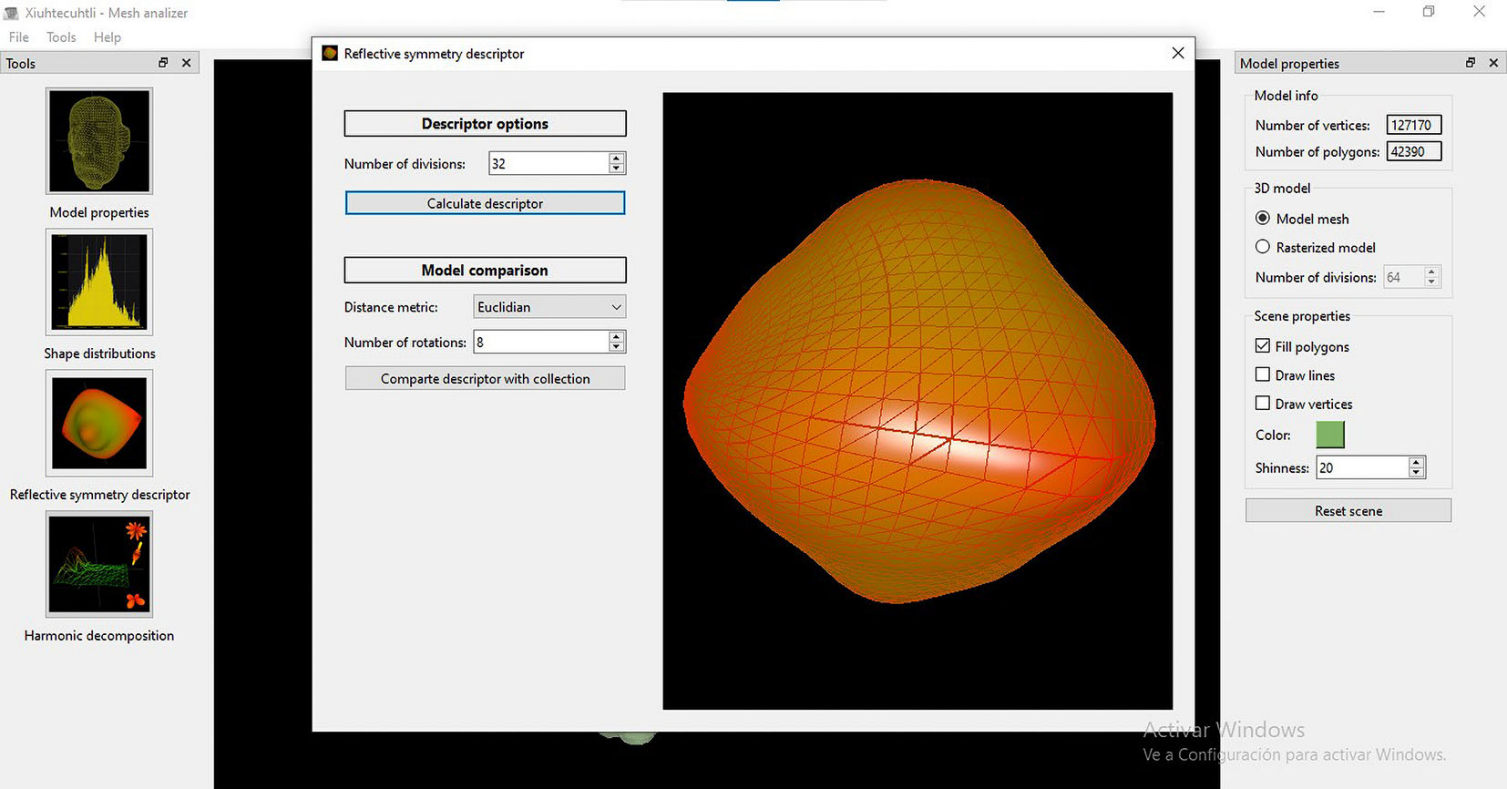
Screenshot of the window to apply the Relective Simmetry algorithm to perform a matching and retrieval operation with MeshAnalizer.

**Figure 13.**
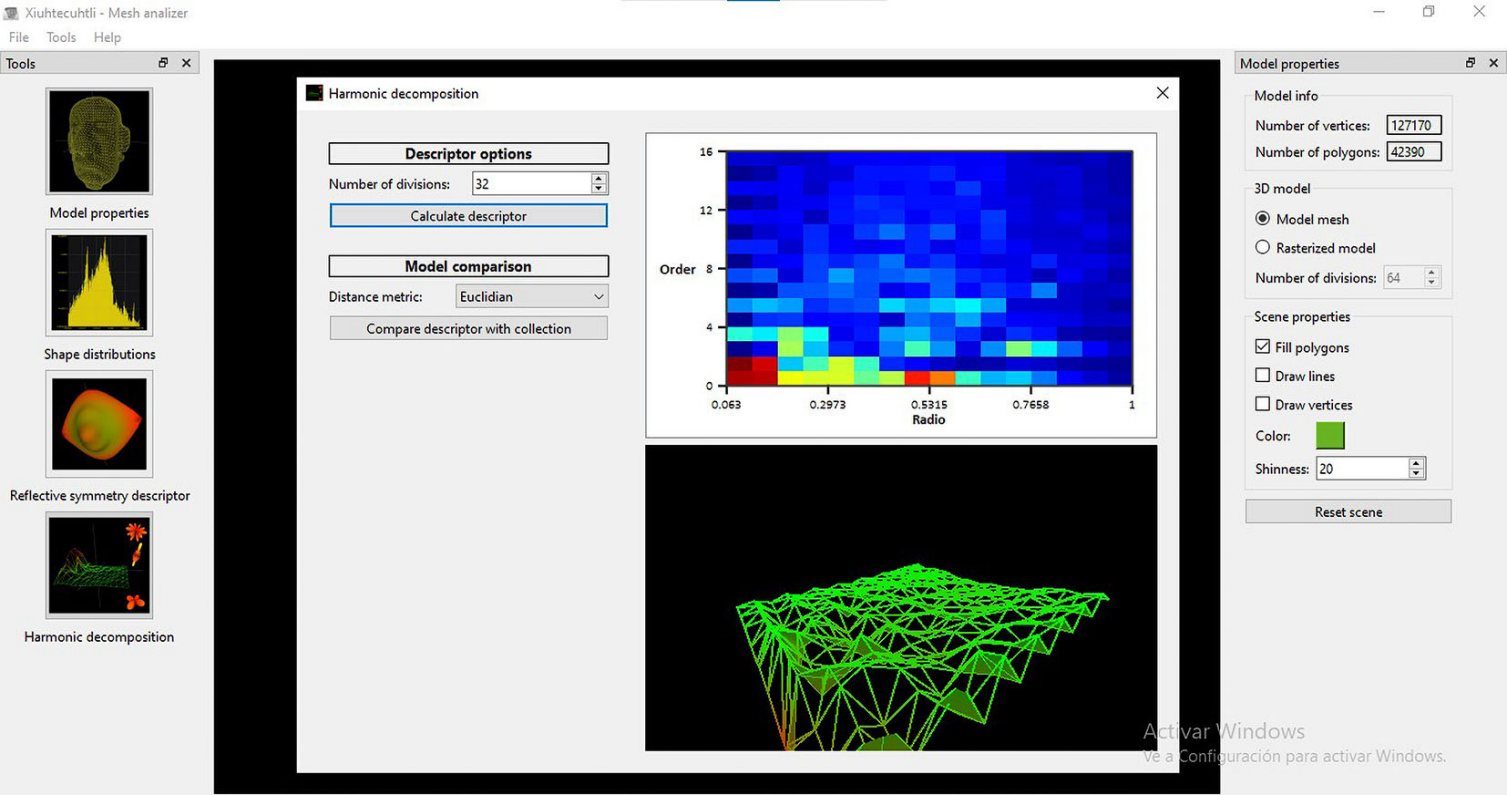
Screenshot of the window to apply the Spherical Harmonics algorithm to perform a matching and retrieval operation with MeshAnalizer.

**Figure 14.**
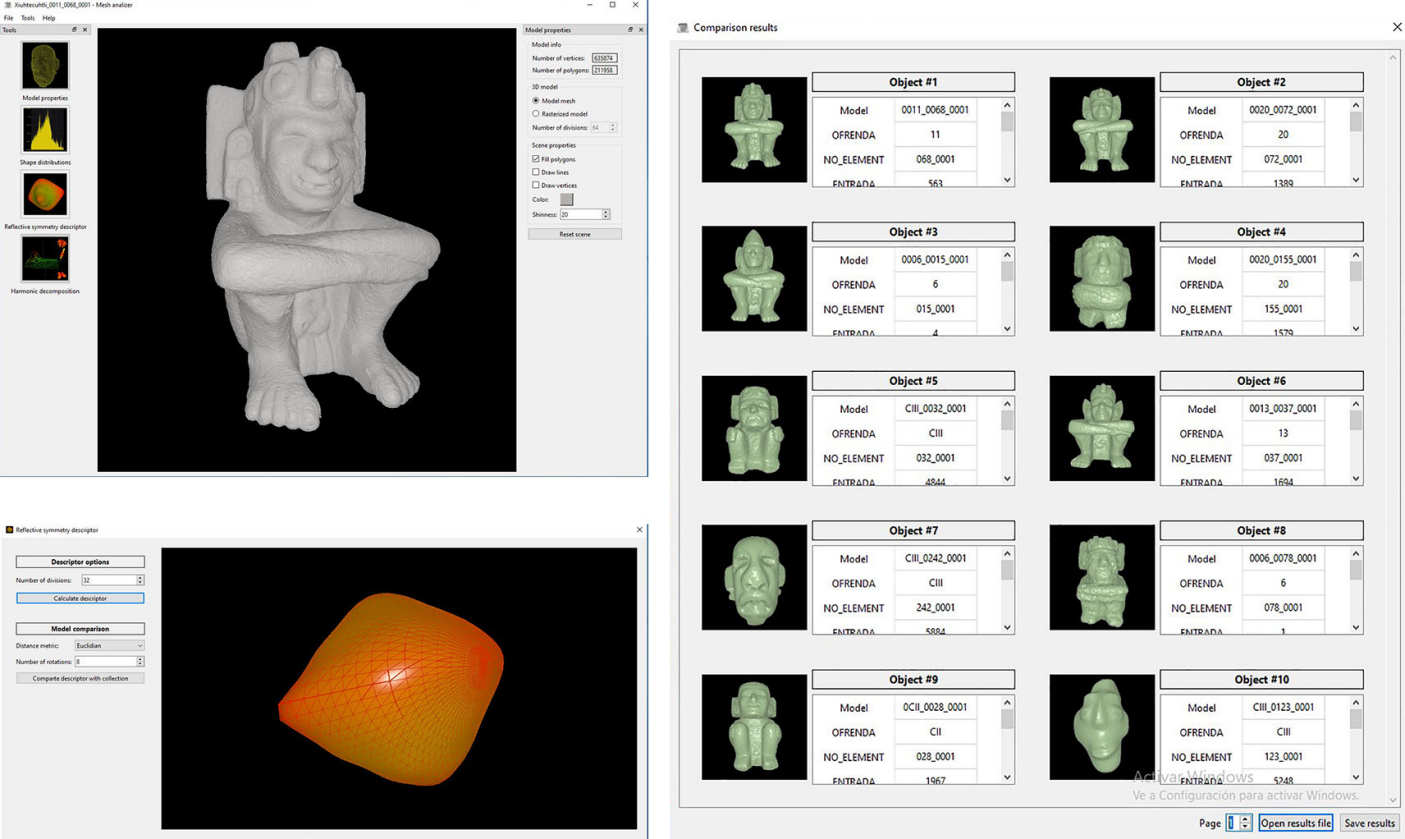
Illustration of the software developed for the search and recovery of 3D models of archaeological objects. Above, on the left, the consultation model ( *i.e.* anthropomorphic sculpture) is shown; below left illustrates obtaining the reflexive symmetry descriptor for that query model; on the right are the search results obtained when the user requests to compare the query model with the models stored in the repository. It can be seen that the system retrieves all objects similar to the query model.

**Figure 15.**
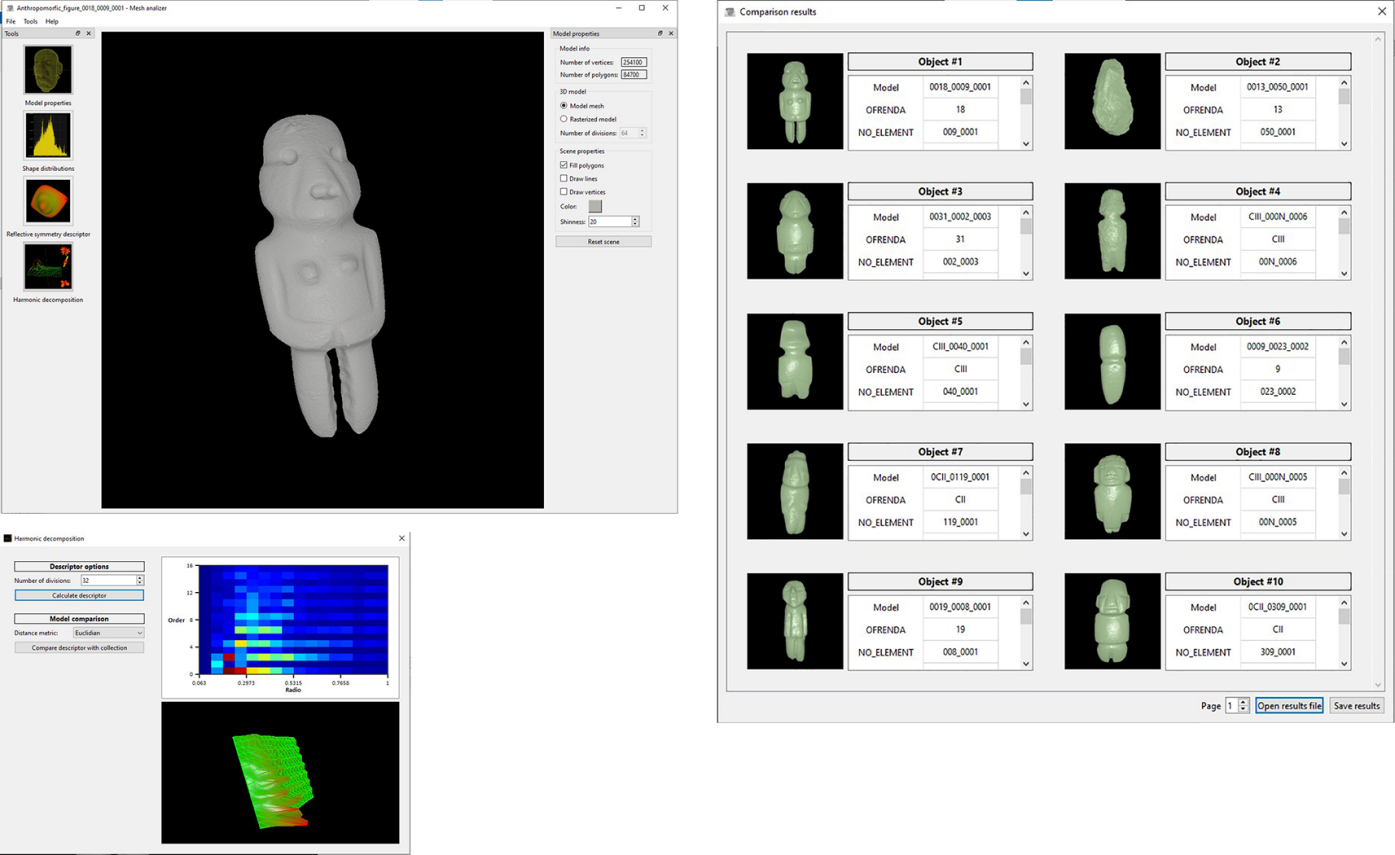
Another example of searching and retrieving 3D models. In this case, all copies of anthropomorphic figures were requested from the system applying the descriptor of harmonic functions.

**Figure 16.**
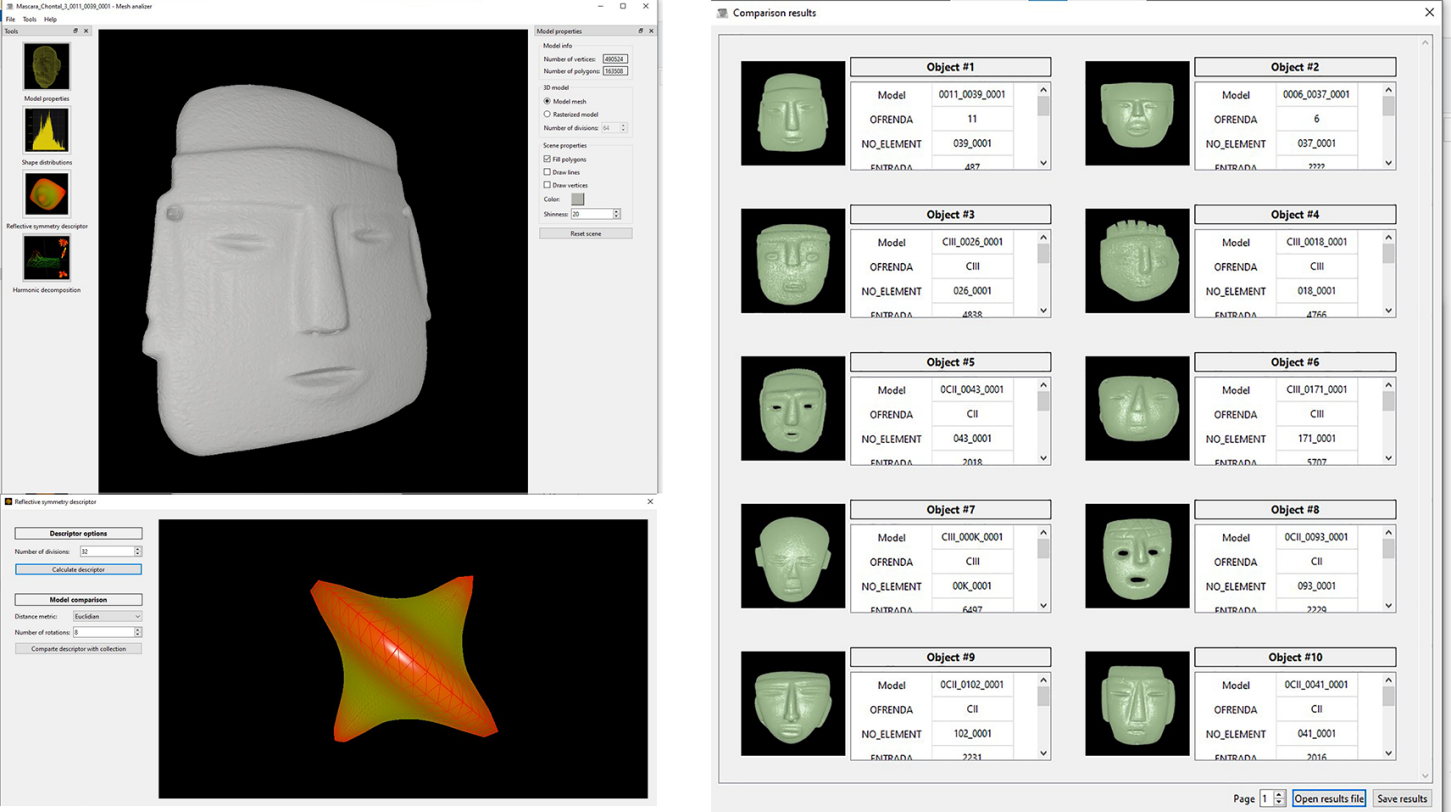
A third example of how the software works. In this case stone masks were recovered. It should be noted that, despite the fact that the query model lacks a fragment, the system was able to produce the expected results, even recovering a mask fragment that clearly belongs to the class of the objects that the user expected.

• A typical operation starts when the user opens a file corresponding to a query 3D model. This is a point cloud or surface mesh that corresponds to the class of object that the user wants to use as example to retrieve all similar objects from the database ( Figure 8). This type of content-based query, makes it unnecessary to input text for the search and matching operations.
• Once the model has been loaded, it is possible to open a window to display model properties such as number of vertices and faces ( Figure 9). The user can choose to render the query model as a triangular mesh or as a rasterized ( *i.e.* voxelized) model ( Figure 10). Additional options include displaying the query model as a solid surface, a triangular mesh, or as a point cloud. From the same window, the colour and level of shininess can also be adjusted.
• Once the model is loaded in the MeshAnalizer module, the user can choose any of the three algorithms available ( *i.e.* Shape Histograms, Reflective Symmetry or Spherical Harmonics) to obtain the descriptor for that particular query model. There are no rules to select an algorithm, it is expected that the user tries different options to see which one works best for retrieving a specific set of archaeological objects.
• For each descriptor, the user is presented with some parameters that can be adjusted to obtain a more or less precision in the computation of the shape descriptor. In the case of Shape Distributions ( Figure 11), the user can define how many samples and bins can be used to build the five shape histograms ( *i.e.* distances to centroid, distances between points, area of triangles, volume of tetrahedra or angles between vectors) for that particular query model. The default is 1,048,576 samples and 1,024 bins. The more samples the more accurate the shape representation would be. These histograms are used later to compare the query model with the collection of descriptors of the 3D models stored in the database during the matching and retrieval operation.
• In the case of Reflective Symmetry ( Figure 12) and Spherical Harmonics decomposition ( Figure 13), the user can define the number of divisions - in the X, Y, and Z axes - that will be considered during the voxelization of the model. As mentioned above, both descriptors are computed from this voxelized representation. For example, if this parameter is set to the default value of 32, then the algorithm will divide the mesh into 32 x 32 x 32 parts. The more divisions the more accurate the computation of the descriptor.
• An additional parameter, number of rotations, is available for the computation of reflective symmetry. This refers to how many times the model is rotated to test its symmetry. For every rotation, a plane cuts the model into two halves that are compared by the algorithm to assess how similar ( *i.e.* symmetrical) they are. The descriptor for the object is obtained by concatenating the symmetry measures of all rotations. A large number of rotations would improve the accuracy of the shape descriptor, but it involves higher computational costs. The default value of 8 is considered appropriate for all models.
• The next step is to compare the descriptor of the query model to all the objects’ descriptors stored in the database ( *i.e.* matching operation). A parameter allows selecting one of the four distance measures implemented, namely Euclidean, City block, Chebychev or Minimun Coordinate.
• Finally, the user presses “Compare descriptor with collection” and the search module proceeds to compare the query model with the descriptors of the models stored in the database, retrieving the results, which are shown in a new window. There is no limit on the number of results displayed by the module. The interface allows saving the results for future reference. Figures 14, 15 and 16 illustrate the user interface during three query examples.

## Conclusions

Computer tools for shape matching and retrieval designed specifically for archaeological research could improve access to collections in museum institutions. The development of the module presented here is a step forward in this direction.

The search-engine module developed over this project is generic, so we expect they would prove helpful in other contexts. The capacity of our system to perform matching and retrieval of real archaeological objects through the application of shape distributions, reflective symmetry, and spherical harmonics descriptors is significant. However, an extra module to provide full database capabilities to store, update and edit the 3D models are still under construction. Also, we expect to perform a benchmark analysis, whose results will be published shortly.

Particularly important for further development is the implementation of additional shape descriptors that target local features, since these would help to refine the queries to specific details on the objects geometry.

In such endeavor we intent to take advantage of the experience from colleagues in Computer Vision. Attene *et al.* (2009), for example, have develop a pipeline - and a software called ShapeAnnotator, which segments 3D meshes into parts that are then combined to form meaningful features. The system annotates the resulting features according to an ontology. Concepts in the ontology are entities with meaning that final users can identify and select in an intuitive interface. Furthermore, by analysing the topology and geometry of the segmented parts, the system can relate the features of one type of object to similar instances stored in a knowledge base. These pipeline and software have been applied to recognized parts of virtual avatars and manufacturing parts, but the framework can be used in other domains thanks to its independence of the geometry of the models and the domain ontology. Thus, we would consider this work for future development of ArcheoShape.

We plan to embed the search-engine module described here into a web platform which will be organized around three main application channels:

1. The first channel would be a service platform for the automatic recognition, analysis, and classification of cultural heritage objects based on morphology. Any user can upload a collection of 3D models to have it analysed with the software tools developed throughout the project. For this operation, the user will not need any knowledge of Computer Vision or Machine Learning because all necessary software will be accessible through a very easy-to-use interface.
2. The second channel called research will be designed to encourage specialized collaboration between experts in Computer Vision, Machine Learning, and shape analysis interested in developing new algorithms, applications, and tools for morphological analysis of cultural heritage. Including our current deep learning applications for shape analysis and retrieval. This collaboration will facilitate access to papers, project proposals, discussion forums, and source code. New solutions to technical problems will be expected to evolve from this site. For example, one pervasive challenge when applying machine learning to archaeology is the lack of enough data to train automatic learning models. This channel could provide a forum for discussing new solutions, such as conditions for applying transfer-learning techniques to train models with external knowledge.
3. The third channel will be named People Interaction. Through this channel, scholars, students, and anyone interested in the project can establish collaboration for future projects and share data and resources from all over the world. The main objective is to create synergy to facilitate access to new 3D digital collections and to define new initiatives of morphological analysis with applications to archaeology and the Humanities.

## Data Availability

Zenodo: omendoza83/ArcheoShape-Data: ArcheoShape 0.2. https://doi.org/10.5281/zenodo.7591490 ( Mendoza-Montoya, 2023a). This project contains the following underlying data:
• Models. (10 triangular meshes of Aztec objects). • Resources. (6983 numerical shape descriptors, computed from 495 archaeological objects). • Icons. (Images for the user interface). • Screenshots. (Images of 495 archaeological objects, used to present results at the end of a search and matching operation). Data are available under the terms of the Creative Commons Attribution-NonCommercial-NoDerivatives 4.0 International (CC-BY-NC-ND 4.0).

## Footnotes

^1^
Also known as the L∞ metric, Chevychev distance determines which distance between two vectors is the greatest of their differences along any coordinate dimension.

^2^
The voxel grid preserves the topological characteristics of the original 3D model. These include connectivity, separation, coverage, and tunnelling. Connectivity registers the way voxels must be linked to each in order to preserve the specific shape of the object. Separation registers empty spaces and how these interact in the voxelised object. Coverage, register how “thin” or thick” the voxelised object is. Tunnelling registers the penetration of two voxelised elements of the object, that is how two or more parts of the object overlap and/or penetrate into each other ( Aleksandrov *et al*., 2021).

^3^
The authors can provide details of the legal and administrative procedures involved in sharing the collection.

## Software availability

Source code available from: https://github.com/omendoza83/ArcheoShape/tree/v0.3.0-alpha

Archived source code at the time of publication: https://doi.org/10.5281/zenodo.7583722 ( Mendoza-Montoya, 2023b)

License: MIT
