# Supplementary material for: Application of computer vision techniques for 3D matching and retrieval of archaeological objects
Source: F1000Res. 2024 Mar 25;12:182. Originally published 2023 Feb 16. [Version 2] doi: 10.12688/f1000research.127095.2 (PMC11359975; doi:10.12688/f1000research.127095.2)
Supplement: Peer review history

# Reviewer report: Reviewer response for version 2

**DOI:** 10.5256/f1000research.163763.r258907

**Published:** 2024-08-28

**Authors:** Federica Maietti

**Affiliations:** 1 Department of Architecture, University of Ferrara, Ferrara, Italy

No comments

Are the conclusions about the tool and its performance adequately supported by the findings presented in the article?

Yes

Is the rationale for developing the new software tool clearly explained?

Yes

Is the description of the software tool technically sound?

Partly

Are sufficient details of the code, methods and analysis (if applicable) provided to allow replication of the software development and its use by others?

Partly

Is sufficient information provided to allow interpretation of the expected output datasets and any results generated using the tool?

Yes

Reviewer Expertise:

Digital Heritage; 3D Survey; Digital Representation; 3D Modeling; Diagnostic procedures; Heritage assessment

I confirm that I have read this submission and believe that I have an appropriate level of expertise to confirm that it is of an acceptable scientific standard.

# Reviewer report: Reviewer response for version 2

**DOI:** 10.5256/f1000research.163763.r258908

**Published:** 2024-04-04

**Authors:** Edgar Roman-Rangel

**Affiliations:** 1 Computer Science Department, Instituto Tecnológico Autónomo de México, Mexico City, Mexico

Author have improved their paper and attending to my previous revision. The new version defines its contribution much clearer, and it has sections that are much better defined.

 I recommend that this version is acceptable.

Are the conclusions about the tool and its performance adequately supported by the findings presented in the article?

Yes

Is the rationale for developing the new software tool clearly explained?

Yes

Is the description of the software tool technically sound?

Partly

Are sufficient details of the code, methods and analysis (if applicable) provided to allow replication of the software development and its use by others?

No

Is sufficient information provided to allow interpretation of the expected output datasets and any results generated using the tool?

Yes

Reviewer Expertise:

Machine learning; Computer vision; Representation learning; Computational archaeology.

I confirm that I have read this submission and believe that I have an appropriate level of expertise to confirm that it is of an acceptable scientific standard.

# Reviewer report: Reviewer response for version 1

**DOI:** 10.5256/f1000research.139568.r200597

**Published:** 2023-09-22

**Authors:** Federica Maietti

**Affiliations:** 1 Department of Architecture, University of Ferrara, Ferrara, Italy

The article is focused on 3D digitization of heritage artefacts and archaeological collections, and on the increasing need for developing means to access the resulting 3D models. In particular, the paper explores shape recognition techniques, deepening three shape descriptors (distributions, reflective symmetry and spherical harmonics) as part of the development of a search engine that retrieves 3D models from an archaeological database.

 Aztec archaeological objects belonging to the Templo Mayor Museum, in Mexico City, are the use case proposed to support the tool description, explanation and applicability.

 The topic is very relevant nowadays, since main challenges and research directions are oriented toward an improvement in robustness and efficiency of the 3D digitization process, also for collections of objects, increasing the accuracy and completeness of surface appearance, and increasing data sharing, dissemination and usability. Retrieve 3D models from large collections is a curatorial and research need to be addressed.

 Possible uses in other contexts and for other collections are given due consideration, and the search-engine module is expected to be tested other contexts.

 Future developments are correctly specified: an extra module to provide full database capabilities to store, update and edit the 3D models is foreseen; a benchmark analysis will be delivered; the implementation of additional shape descriptors helping to refine queries is mentioned as well.

 Some minor comments:

- The State of the Art concerning heritage digitization via photogrammetry (photomodelling / Structure from Motion) and laser scanning can be improved, as well as data segmentation and classification. The statement: “Currently, the conventional way to locate models in a database consists of formulating a query by using keywords that describe the objects’ features” does not consider latest innovations in semantic web.
  In general, references on Machine Learning and new avenues in shape recognition can be improved. Just some suggestions:
https://doi.org/10.5194/isprs-archives-XLVIII-M-2-2023-1359-2023
https://doi.org/10.3390/rs11070847
- The workflow for the implementation of the ArcheoShape search-engine module is clearly described, focusing on comparing computational shape descriptions. Anyway, tool “architecture” should be described in more detail, in order to provide means for replication of the software development by other users.
- Expanded query criteria should be listed and explained.

Are the conclusions about the tool and its performance adequately supported by the findings presented in the article?

Yes

Is the rationale for developing the new software tool clearly explained?

Yes

Is the description of the software tool technically sound?

Partly

Are sufficient details of the code, methods and analysis (if applicable) provided to allow replication of the software development and its use by others?

Partly

Is sufficient information provided to allow interpretation of the expected output datasets and any results generated using the tool?

Yes

Reviewer Expertise:

Digital Heritage; 3D Survey; Digital Representation; 3D Modeling; Diagnostic procedures; Heritage assessment

I confirm that I have read this submission and believe that I have an appropriate level of expertise to confirm that it is of an acceptable scientific standard, however I have significant reservations, as outlined above.

## References

[1] : AN APPROACH TO BUILD A COMPLETE DIGITAL REPORT OF THE NOTRE DAME CATHEDRAL AFTER THE FIRE, USING THE AIOLI PLATFORM. *The International Archives of the Photogrammetry, Remote Sensing and Spatial Information Sciences* .2023;XLVIII-M-2-2023: 10.5194/isprs-archives-XLVIII-M-2-2023-1359-2023 1359-1365 10.5194/isprs-archives-XLVIII-M-2-2023-1359-2023

[2] : Classification of 3D Digital Heritage. *Remote Sensing* .2019;11(7) : 10.3390/rs11070847 10.3390/rs11070847

# Response

**Published:** 2024-03-13

**Authors:** Diego Jiménez-Badillo

**Affiliations:** Instituto Nacional de Antropología e Historia (INAH), Mexico

Thanks for your comments and suggestions, which have allowed us to improve this manuscript by doing the following changes:

- Reviewer comment: The State of the Art concerning heritage digitization via photogrammetry (photomodelling / Structure from Motion) and laser scanning can be improved, as well as data segmentation and classification. The statement: “Currently, the conventional way to locate models in a database consists of formulating a query by using keywords that describe the objects’ features” does not consider latest innovations in semantic web. In general, references on Machine Learning and new avenues in shape recognition can be improved. **Response**: We extended the Introduction with descriptions and references on photomodelling / Structure from Motion, as well as advances in Semantic Web and deep-learning for accessing cultural heritage information, particularly the use of ontologies, as well as segmentation and annotation techniques. We also added comments on the papers that you recommended.
- The second reviewer also considers that the paper only describes the software tool partly and that there are no enough details of the code, method and analysis. **Response**: We offer the same response given to Reviewer 1: Regarding the methods and analysis, we consider that these are sufficiently covered in the explanation of the three descriptors implemented, where the principles, methods and analysis of each algorithm are explained. As for the tool itself, we included more details of MeshAnlizer architecture, as well as a lengthy explanation of how it functions. As for a technical description of the source code, we consider that as the implementation of the whole system is still a work in progress and likely to change over time, readers that want more details -or want to replicate the software- are better served by documenting the source code with commentaries on functionality directly on the source code. This is available in the GitHub repository, from which anyone can download the source code to compile it as such, adapt it or replicate it freely for his own purposes. Documenting the software in this manner, would guarantee that as the module changes the users can always get an explanation of the latest progress.

# Reviewer report: Reviewer response for version 1

**DOI:** 10.5256/f1000research.139568.r163745

**Published:** 2023-02-24

**Authors:** Edgar Roman-Rangel

**Affiliations:** 1 Computer Science Department, Instituto Tecnológico Autónomo de México, Mexico City, Mexico

This work presents a software tool that implements a retrieval engine for 3D models of archaeological nature. The retrieval machine combines several shape descriptors and distance functions to obtain different ways of indexing the 3D models.

 The need for this tool is well motivated, and the results shown in the document seem both correct and relevant. Moreover, without a doubt the tool is an interesting application and will constitute an asset for improving the work of archaeologists.

 However, there is one details that need to be addressed. Concretely, the document focusses more on the mathematical details of the 3D shape descriptors, rather than focussing on the implementation details of the tool itself. Improving this aspect of the presentation will help fit the paper into the scope of the journal.

 Additionally, there a few minor observations:

- The fourth paragraph of the introduction states "...analysing the shape of the objects without relying exclusively on keywords...". The term for that concept is Content-based Information Retrieval (CBIR).
- "... without being affected by the variations of ...". The term "affine variations" will be more specific.
- The section named "Development of a search-engine module" reads more like related work, and not like the description of the search engine.
- The third paragraph of page 5, says that there are thirty combinations of descriptors-distances, and makes reference to Table 1. However, Table 1 only shows the different distances.
- The functions D(f (x), sg (sx)), in page 6 are not defined.

Are the conclusions about the tool and its performance adequately supported by the findings presented in the article?

Yes

Is the rationale for developing the new software tool clearly explained?

Yes

Is the description of the software tool technically sound?

Partly

Are sufficient details of the code, methods and analysis (if applicable) provided to allow replication of the software development and its use by others?

No

Is sufficient information provided to allow interpretation of the expected output datasets and any results generated using the tool?

Yes

Reviewer Expertise:

Machine learning; Computer vision; Representation learning; Computational archaeology.

I confirm that I have read this submission and believe that I have an appropriate level of expertise to confirm that it is of an acceptable scientific standard, however I have significant reservations, as outlined above.

# Response

**Published:** 2024-03-13

**Authors:** Diego Jiménez-Badillo

**Affiliations:** Instituto Nacional de Antropología e Historia (INAH), Mexico

Reviewer comment: "The fourth paragraph of the introduction states "...analysing the shape of the objects without relying exclusively on keywords...". The term for that concept is Content-based Information Retrieval (CBIR)."

**Response**: We added the term Content-based Information Retrieval (CBIR) in the corresponding paragraph of the Introduction.

 Reviewer comment: The section named "Development of a search-engine module" reads more like related work, and not like the description of the search engine.

**Response**: We added more details of the MeshAnalizer module, including a new figure illustrating its architecture.

 Reviewer comment: "The third paragraph of page 5, says that there are thirty combinations of descriptors-distances, and makes reference to Table 1. However, Table 1 only shows the different distances."

**Response**: We moved the Table 1 reference immediately after the mention of the four distance measures implemented. This would avoid confusion on the kind of information that reader may expect in Table 1. 

 Reviewer comment: "The functions D(f (x), sg (sx)), in page 6 are not defined."

**Response**: We defined the functions.

 Also, the reviewer considers that the paper only describes the software tool partly and that there are no enough details of the code, method and analysis.  

**Response**: Regarding the methods and analysis, we consider that these are sufficiently covered in the explanation of the three descriptors implemented, where the principles, methods and analysis of each algorithm are explained. As for the tool itself, we included more details of MeshAnlizer architecture, as well as a lengthy explanation of how it functions. We believe that this improves the section “Development of a Search Engine Module”.
